# Characterizing mRNA Interactions with RNA Granules during Translation Initiation Inhibition

**DOI:** 10.1371/journal.pone.0019727

**Published:** 2011-05-05

**Authors:** Chiara Zurla, Aaron W. Lifland, Philip J. Santangelo

**Affiliations:** Wallace H. Coulter Department of Biomedical Engineering, Georgia Institute of Technology and Emory University, Atlanta, Georgia, United States of America; Victor Chang Cardiac Research Institute (VCCRI), Australia

## Abstract

When cells experience environmental stresses, global translational arrest is often accompanied by the formation of stress granules (SG) and an increase in the number of p-bodies (PBs), which are thought to play a crucial role in the regulation of eukaryotic gene expression through the control of mRNA translation and degradation. SGs and PBs have been extensively studied from the perspective of their protein content and dynamics but, to date, there have not been systematic studies on how they interact with native mRNA granules. Here, we demonstrate the use of live-cell hybridization assays with multiply-labeled tetravalent RNA imaging probes (MTRIPs) combined with immunofluorescence, as a tool to characterize the polyA+ and β-actin mRNA distributions within the cytoplasm of epithelial cell lines, and the changes in their colocalization with native RNA granules including SGs, PBs and the RNA exosome during the inhibition of translational initiation. Translation initiation inhibition was achieved via the induction of oxidative stress using sodium arsenite, as well as through the use of Pateamine A, puromycin and cycloheximide. This methodology represents a valuable tool for future studies of mRNA trafficking and regulation within living cells.

## Introduction

When cells are exposed to an assortment of environmental stresses, global translational arrest of housekeeping transcripts is accompanied by the formation of distinct cytoplasmic structures known as stress granules (SGs) and an increase in the number of p-bodies (PBs) [1], [2]. The core constituents of SGs are components of a noncanonical, translationally silent 48S pre-initiation complex that includes the small ribosomal subunit and early initiation factors eIF4E, eIF3, eIF4A, eIFG and PABP. SGs also contain mRNAs and a set of mRNA binding proteins that regulate mRNA translation and decay, as well as proteins that regulate various aspects of mRNA metabolism [3], [4]. PBs consist of a core of proteins involved in mRNA repression and degradation, including the mRNA decapping machinery [5], as well as key effectors of microRNA (miRNA)-mediated RNA interference (RNAi), such as Argonaute-2 (Ago2), miRNAs, and their cognate mRNAs [6]. Given their protein content, these cytoplasmic foci are thought to represent key players in the regulation of translation. Specifically, SGs are considered aggregates of translationally inactive mRNAs containing stalled translation initiation complexes while PBs are considered sites of mRNA decay and storage containing the 5′-to-3′ decay enzymes and activators. While SGs and PBs have been extensively studied from the perspective of their protein content and dynamics and progress has been made in understanding their role in translational repression, the study of native mRNA dynamics during translational inhibition has been limited by the difficulty with detecting native mRNA with single RNA sensitivity. mRNA localization within SGs and PBs during stress has been inferred using fluorescence microscopy mainly in three ways i) directly using *in situ* hybridization (FISH) with immunofluorescence, or ii) using plasmid-derived mRNA systems and iii) indirectly, by monitoring the behavior of known binding proteins such as TIAR/-1 or PABP [7]. FISH has been successfully combined with immunofluorescence [8], but its general applicability is limited by the effects of chemicals such as formamide on epitopes and antibody binding. In addition, FISH does not allow for live-cell studies. Plasmid-derived systems, such as the MS2-GFP system, have extensively been utilized since they do allow for live cell monitoring, however they can suffer from complicated stochiometric effects due to overexpression of transcripts. Other options for the study of native mRNA are therefore required to gain both dynamic potential and single molecule sensitivity.

We recently reported the use of multiply labeled tetravalent imaging probes (MTRIPs) for imaging native, non-engineered RNA in live cells with single molecule sensitivity. These probes consist of four linear oligonucleotides labeled with multiple fluorophores, bound together by the biotin-streptavidin linkage. MTRIPs, when delivered to cells by streptolysin-O reversible permeabilization, recognize their intracellular target through Watson-Crick base pairing; signal is raised above background by the binding of at least 2 probes per target RNA [9]. We previously used MTRIPs to successfully target β-actin and Arp2 mRNAs, as well as viral genomic RNA, in epithelial cells and primary chicken fibroblasts. MTRIPs were also used to demonstrate viral RNA/SG interactions [9].

Here, we demonstrated the coupling of live-cell delivery of MTRIPs with immunofluorescence in order to quantify the colocalization of mRNAs with SGs and PBs, and the changes in their interactions in the presence of different treatments such as sodium arsenite, Pateamine A, puromycin and cycloheximide, which have been shown to influence SGs and/or PBs formation and dynamics [10]. We either targeted β-actin mRNAs or a general set of mRNAs by using probes targeted against polyA+ RNA. β-actin mRNA was chosen, specifically, because its localization has been extensively investigated as a function of its transcriptional/translational state, as well as during the stress response [11], [12], [13], [14]. Moreover, β-actin mRNA have been shown to interact with proteins that play a crucial role during the stress response, such as TIA-1 [15], and with proteins involved in localization and stabilization of mRNA, such as HuR and ZBP1 [11], [16]. To demonstrate MTRIPs versatility, we performed our experiments using U2OS and DU145 cells, since they have been widely used to study the stress response [10], [13] as well as A549, where the accuracy of MTRIPs targeting mRNAs was characterized for the first time [9].

In addition, fluorescence microscopy using MTRIPs allowed us to observe that, upon translation initiation inhibition, a fraction of mRNA granules consistently localized near the nucleus, where they coalesced into large perinuclear aggregates excluded from SGs or PBs, similarly with what was previously observed in oligodendrocytes by Thomas and colleagues [17]. Delivery of MTRIPS targeting polyA+ as well as β-actin mRNAs demonstrated that these transcripts localized near the microtubule organizing center (MTOC), with a mechanism independent on eIF2a phosphorylation but dependent on an intact microtubule network, as observed using pateamine A and nocodazole, respectively. Staining for the exosomal subunits RRP40 and RRP41, showed clear colocalization with the mRNAs near the MTOC, suggesting interactions between the transcripts and the 3′-to-5′ decay machinery. RRP40 and RRP41 were previously shown to form cytoplasmic granules near the nucleus in unstressed HeLa-TO cells [18]. Such granules were shown to contain mRNA decay enzymes including PARN and exosome subunits such as RRP4, RRP40 PM-Scl75, RRP46 and RRP41. These so-called exosome granules did not overlap with PBs and SGs and accumulated a reporter ARE-mRNA, suggesting a role in the AMD (ARE-mediated mRNA decay). Our data confirmed such observations and suggested, for the first time, how the RNA exosome may participate to the stress response.

## Materials and Methods

### Cell lines

A549 (ATCC CCL-185), U2OS (ATCC HTB-96) and DU145 (ATCC HTB-81) cells were cultured in DMEM (Lonza) with 10% FBS (Hyclone), 100 U/ml penicillin, and 100 µg/ml streptomycin (Invitrogen). Cells were plated on glass coverslips one day prior to experiments.

### MTRIPs synthesis and delivery

MTRIPs were synthesized as previously described [9]. Briefly, the 2*′*-O-methyl RNA/DNA chimera nucleic acid ligands (Biosearch Technologies, Inc. Novato, CA) contain a 5*′*-biotin modification and multiple dT-C6-NH_2_, modifications. Probes were assembled by first labeling the free amine groups on the ligands with Cy3B-NHS ester (GE Healthcare) using manufacturer protocols. Free dye was removed using 3 kD Nanosep spin columns (Pall Corp.). The purified ligands were resuspended in 1×PBS and mixed at a 5∶1 molar ratio with neutravidin for 1 hour at RT. Free ligands were removed using 30 kD Nanosep spin columns. When multiple probes were utilized, each probe was assembled separately and then mixed in SLO/medium just prior to delivery in equimolar concentrations. Probe sequences are reported in Table S1. MTRIPs were subsequently delivered into cells using reversible membrane permeabilization. Briefly, 2 U/ml Streptolysin O (SLO) (Sigma) were first reduced using 7.5 mM Tris(2-carboxyethyl) phosphine (TCEP) (Peirce) for 1 hr at 37°C. Cells were rinsed using PBS (−Ca^2+^ −Mg^2+^) (Thermo) and then incubated with delivery medium containing 0.2 U/ml SLO and probes in Optimem (Gibco) for 10 min at 37°C. The delivery medium was then removed and replaced with DMEM for 15 min for recovery. For live cell imaging experiments growth media was replaced with Leibovitz's L15 medium (Invitrogen) immediately prior to image acquisition.

### Immunostaining

After probe delivery, cells were fixed with 4% paraformaldehyde (Electron Microscopy Science) in PBS, permeabilized using 0.2% triton-X 100 (Sigma), and blocked with 5% bovine serum albumin (Ambion). Cells were then incubated with primary antibodies for 30 min at 37°C and with secondary antibodies for 30 min at 37°C. After DAPI staining (Invitrogen) cells were mounted on slides using Prolong (Invitrogen). For microtubule staining, cells were washed with BRB80 buffer (80 mM Pipes pH 6.8, 1 mM MgCl_2_, 1 mM EGTA) and subsequently fixed in 4% paraformaldehyde in BRB 80 buffer. For γ-tubulin staining, cells were fixed in 100% methanol for 10 min at −20°C and permeabilized using 100% acetone for 2 min at −20°C.

### Antibodies

Primary antibodies were goat anti-TIAR, mouse monoclonal anti-HuR, goat anti-EXOSC3 (RRP40) and rabbit anti-EXOSC4 (RRP41) (Santa Cruz Biotechnology), monoclonal and rabbit anti-G3BP (BD and Sigma respectively), rabbit anti γ-tubulin (Sigma), monoclonal anti α-tubulin (Molecular Probes). Rabbit anti-DCP1α was kindly provided by Dr. Lykke Andersen. Alexa 488 Phalloidin was from Invitrogen. The E7 monoclonal antibody against β-tubulin was developed by M. Klymkowsky was obtained from the Developmental Studies Hybridoma Bank.

### Drugs

Cells were incubated for 1 h at 37°C with 0.5 mM sodium arsenite at unless differently specified, for 30 min with 600 nM nocodazole in U2OS and 3 µM in A549, for 30 min with 10 µg/ml puromycin or cycloheximide, 90 min with 10 µg/ml vinblastin and 30 min with 200 nM Pateamine A. All drugs were from Sigma. Pateamine A was kindly provided by Dr. Pelletier.

### qTR-PCR

Control U2OS cells or cells treated with SLO with and without MTRIPS (ACTB1 and ACTB2 30 nM each) were incubated with 5 µM Actinomycin D (Sigma) and total RNA was extracted at the indicated time points using the RNeasy Mini kit (Quiagen). Total RNA was subsequently checked for integrity via agarose gel electrophoresis and quantified via UV-VIS spectrometry. 1 µg total RNA was used for cDNA synthesis using the RT^2^ first strand kit (SA biosciences) according to the manufacturer instructions. 1 µl of the product was then used for qRT-PCR using the Real-time RT^2^ qPCR primer assay (SYBR green) in the presence of gene-specific primers for ACTB and GAPDH (SA bioscences). qRT-PCR was performed using ABI StepOnePlus real-time PCR system (Applied biosciences).

### Cell transfection

U2OS cells were cotransfected with 1 µg of pACGFP-actin plasmid (Clonetech) and either 50 nM or 200 nM siRNA targeting β-actin mRNA (On target plus smart pool, Dharmacon thermo Scientific) or 30 nM MTRIPs (ACTB 1 and ACTB 2) via electroporation using the Neon system (Invitrogen) according to manufacturer instructions. 48 h post transfection cells were fixed, DAPI stained and mounted. GFP-actin synthesis in each experimental condition was quantified via fluorescence microscopy at a similar exposure time and gain. 200 cells were analyzed in two independent experiments.

### FISH

Cells were first fixed in 4% paraformaldehyde in BRB80 buffer, permeabilized in 70% ethanol overnight, rinsed in PBS three times and incubated for 2 h at 50°C in hybridization buffer (2× SSC, 100 µg BSA, 10% dextran sulfate, 50 µg tRNA, 70% formamide, 50 µg salmon sperm DNA and probes at 1 nM each). Probes were linear Cy3B-labeled nucleic acid ligands described above. After hybridization, cells were rinsed with 2×SSC for 5 min, 2×SSC/10% fomamide for 10 min and in 2×SSC for 5 min. For immunofluorescence, cells were subsequently rinsed 3 times in PBS and stained with primary antibodies for 30 min at 37°C and with secondary antibodies for 30 min at 37°C. After DAPI staining cells were mounted on slides using Prolong.

### Fluorescence imaging and image analysis

Images were taken on a Zeiss Axiovert 200 M microscope with a 63× NA 1.4 Plan Apochromat primary objective and a Hammamatsu ORCA-ER AG camera. Fluorescent filter sets used were Chroma 49002 ET-GFP and 49004 ET-Cy3. A Bioptechs Delta T system and objective heater were used for live cell imaging. All imaging experiments were performed using the Volocity acquisition software (Improvision). Image z-stacks were acquired in 200 nm steps. All images were deconvolved using the iterative or the fast restoration algorithms in Volocity. All images, unless otherwise specified, are extended views, which compress all of the imaging planes in the z direction into one imaging plane.

### Quantification

The volume and the percentage of SGs/PBs occupied by mRNAs were measured using the Volocity software (Improvision, PerkinElmer), which provides tools designed specifically for colocalization analysis. Each cell was analyzed individually as follows. SGs or PBs were initially identified using their SD intensity and size; colocalization between mRNAs and RNA granules was then calculated using the Volocity colocalization tool, after thresholding to remove the background signal. Thresholds were individually set for each cell to minimize the complications in measuring fluorescence intensity across independent samples. The threshold for mRNA signal was set by selecting regions of the cell with no overt mRNA signal, which is more conservative than the threshold of single probes bound to glass. Thresholds for SGs and PBs were set by selecting the lowest values at the edges of the granules. SGs and PBs were further filtered by excluding regions found to be smaller than the size of the point spread function. With this procedure, each SG or PB was identified as an object, defined by a ROI and analyzed individually. The voxel ratio resulting from the colocalization analysis was used to quantify the volume of each SG/PB occupied by mRNA granules in each experimental condition and, as a consequence, the percentage of SGs/PBs interacting with mRNAs.

In order to determine the percentage of mRNA granules interacting with SG or PB, RNA granules were first identified as described above and identified by a ROI. mRNAs in each cell were subsequently identified according to their size and SD intensity as in Santangelo *et al* [9] and combined with the RNA granules using the “exclude-non touching objects” tool in Volocity.

In order to analyze mRNA interactions with RRP40/41 enriched granules, the RRP40/41 granules were initially identified according to their intensity and size (as above) and combined using the “intersect” tool, such that only granules containing both proteins were taken into account for further analysis. mRNAs in each cell were subsequently identified according to their size and SD intensity as described above and combined with the exosome granules using the “intersect” tool to visualize the resulting objects (Figures 10 D and H in the results section).The distribution of mRNAs interacting with RRP40/41 enriched granules was analyzed by visual inspection.

For each experiment we analyzed at least 10 representative cells; experiments were routinely repeated twice. All the intensity profiles used to demonstrate colocalization were generated using the ImageJ (NIH) Color Profiler plugin and plotted in Excel. Statistical significance of data was determined in Signal Plot (Systat) using the one way ANOVA for normal data or Kruskal-Wallis for all other cases. Multiple pairwise comparisons were performed versus the control group only by Bonferroni t-test for normal data with equal variance or Dunn's method for all other cases.

## Results

### Quantification of mRNA interactions with SGs and PBs

**Figure 1:**
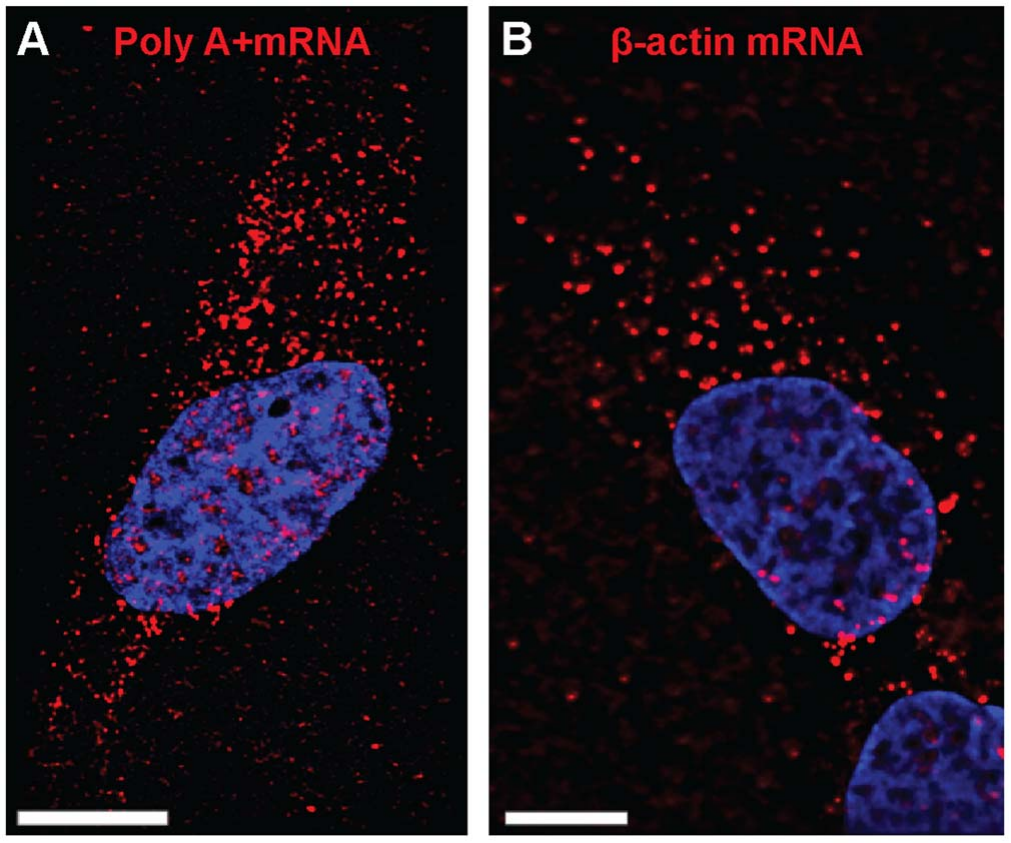
polyA+ and β-actin mRNA distribution. Single plane images showing the distribution of (A) poly A+ and (B) β-actin mRNA granules (red) in controlU2OS cells. Nuclei were stained with DAPI. Scale bars, 10 µm.

We first characterized the distribution of native mRNA granules in untreated U2OS cells by delivering, using streptolysin O (SLO), Cy3B-labeled MTRIPs designed to target either the polyA+ tail of mRNAs or two regions of the human β-actin coding sequence (poly A+ probe 90 nM or ACTB probes 1 and 2, 30 nM each, Figure 1 and Table S1). After delivery, the cells were fixed and various proteins were fluorescently-labeled using immunostaining. Both mRNA populations were found to be distributed in diffraction-limited spots or granules within the cytoplasm, clearly visible in comparison to background noise (Figure S1A, B); as previously shown, β-actin mRNA were abundant in the perinuclear region, in protrusions and along the edges of the cells (Figure 1B and S1B) [9], while poly A+ mRNAs appeared to be relatively more abundant in the perinuclear region (Figure 1A). To further demonstrate MTRIPS specificity we delivered via SLO, simultaneously, probes targeting β-actin mRNA, and a “scrambled” probe, which targets the genomic RNA of respiratory syncytial virus (RSV) at the same concentration, but labeled with a Cy5 equivalent dye, CF640R (Biotium, Inc.) (30 nM, Table S1). As previously demonstrated [9], the latter were distributed in the perinuclear region and in the cytoplasm and did not colocalize with β-actin mRNA (Figures S1C, D and E).

We subsequently investigated if SLO treatment and MTRIPs binding to their target transcripts would affect mRNA stability. While Lloyd *et al.* [19] demonstated that SLO does not affect TNFα mRNA level and protein synthesis, mRNA decay in the presence of targeting MTRIPS has not been tested. To do this, we extracted total RNA from U2OS cells exposed to Actinomycin D for 0-4-8 and 24 h after either a mock treatment, SLO treatment or SLO-mediated MTRIPs delivery. We converted the total RNA in cDNA in the presence of random hexamers and analyzed the reaction product via qRT-PCR in the presence of either ACTB or GAPDH gene specific primers. We used GAPDH as an internal control since it was previously employed to study off target effects of SLO-delivered antisense RNAs [20]. The results, reported as the fold change of ACTB mRNA expression normalized to the control experiment (time 0, no treatment) relative to that observed for GAPDH, demonstrated that neither SLO treatment nor MTRIPS significantly affect mRNA stability for periods up to 8 h post delivery (Figure S2A).

We also studied the effects of MTRIPs on mRNA translatability. To do so, we monitored the expression of GFP-β-actin protein in transiently transfected U2OS cells in the presence of siRNA or MTRIPS, as described in Material and Methods. The results, summarized in Figure S2B, clearly demonstrated that, while siRNAs efficiently lowered the expression of GFP-β-actin, MTRIPs did not inhibit mRNA translation.

**Figure 2:**
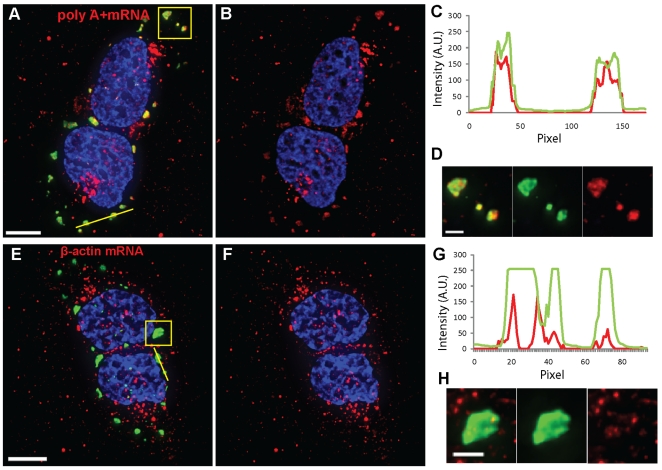
poly A+ and β-actin mRNAs interact with SGs in the presence of sodium arsenite. (A, B) Poly A+ and (E, F) β-actin mRNA granules (red) interact with G3BP-stained SGs (green) in U2OS cells as demonstrated by intensity profiles along yellow lines (C, G) and insets displaying magnification of boxed areas (D,H). β-actin mRNA in B and F and in insets was contrast enhanced to allow visualization of granules. Nuclei were stained with DAPI. Scale bars, 10 µm and inset scale bars, 2.5 µm.

We verified that cell exposure to SLO does not cause SG formation (data not shown), then, after delivering MTRIPs, we induced oxidative stress, by incubating U2OS cells with 0.5 mM sodium arsenite for 1 h at 37°C, the typical sub-lethal concentration used to study stress-dependent translational inhibition. We observed that SGs formed within 90% of the cells and, as expected, they contained TIAR, G3BP and HuR (Figure S3A). SLO treatment had no effect on the efficiency of SG formation or protein content (Figure S3B and C). Using the Volocity software we identified the mRNA granules and the SGs as described in Material and Methods, and quantified their interactions. Poly A+ mRNA granules interacted with all detected SGs and occupied approximately 96% of their volume (Figure 2A, B and C and Table 1). β-actin mRNA granules interacted with over 90% of the analyzed SGs and occupied approximately 52% of their volume (Figure 2D, E and F and Table 1). As can be observed by comparing Figures 2B and F, the polyA+ transcripts within the SGs were larger and more visible than the β-actin ones and clearly filled the SG volume. Similar results were obtained using sodium arsenate at 2.5 mM for 1 h at 37°C in U2OS and treating U2OS cells with 1 mM and 2 mM sodium arsenite for 1 hour at 37°C (Table S2 and data not shown). In this case, while the number of cells containing SGs increased to 100%, SG occupation by β-actin mRNAs was similar to that observed in the presence of 0.5 mM sodium arsenite or slightly lower, indicating that SG occupancy did not increase with the amount of stress, possibly due to saturation.

**Table 1:** SG occupancy by mRNAs under different experimental conditions and cell lines.

			As	Pat A	As+Puro	As+Cyclo	As+Noc
U2OS	ACTB	%SGs	93	69	98	97	79
		% SG vol	52±20	21±9*	87±12*	76±14	34±20
	polyA+	%SGs	100	73	100	100	/
		% SG vol	96±6	25±12*	89±9	61±24*	/
A549	ACTB	%SGs	96	/	98	76	81
		% SG vol	31±14	/	63±14*	48±21	39±16
DU145	ACTB	%SGs	86	/	93	66	/
		% SG vol	20±6	/	29±13	22±11	/

Percentage of SGs (% SGs) and percentage of SG volume (% SG vol) occupied by mRNA granules in all the analyzed experimental conditions and cell lines. Standard deviations values are indicated and

*represents statistically significant difference.

As = sodium arsenite, Pat A = pateamine A, Puro = Puromycin, Cyclo = cycloheximide and Noc = nocodazole.

**Figure 3:**
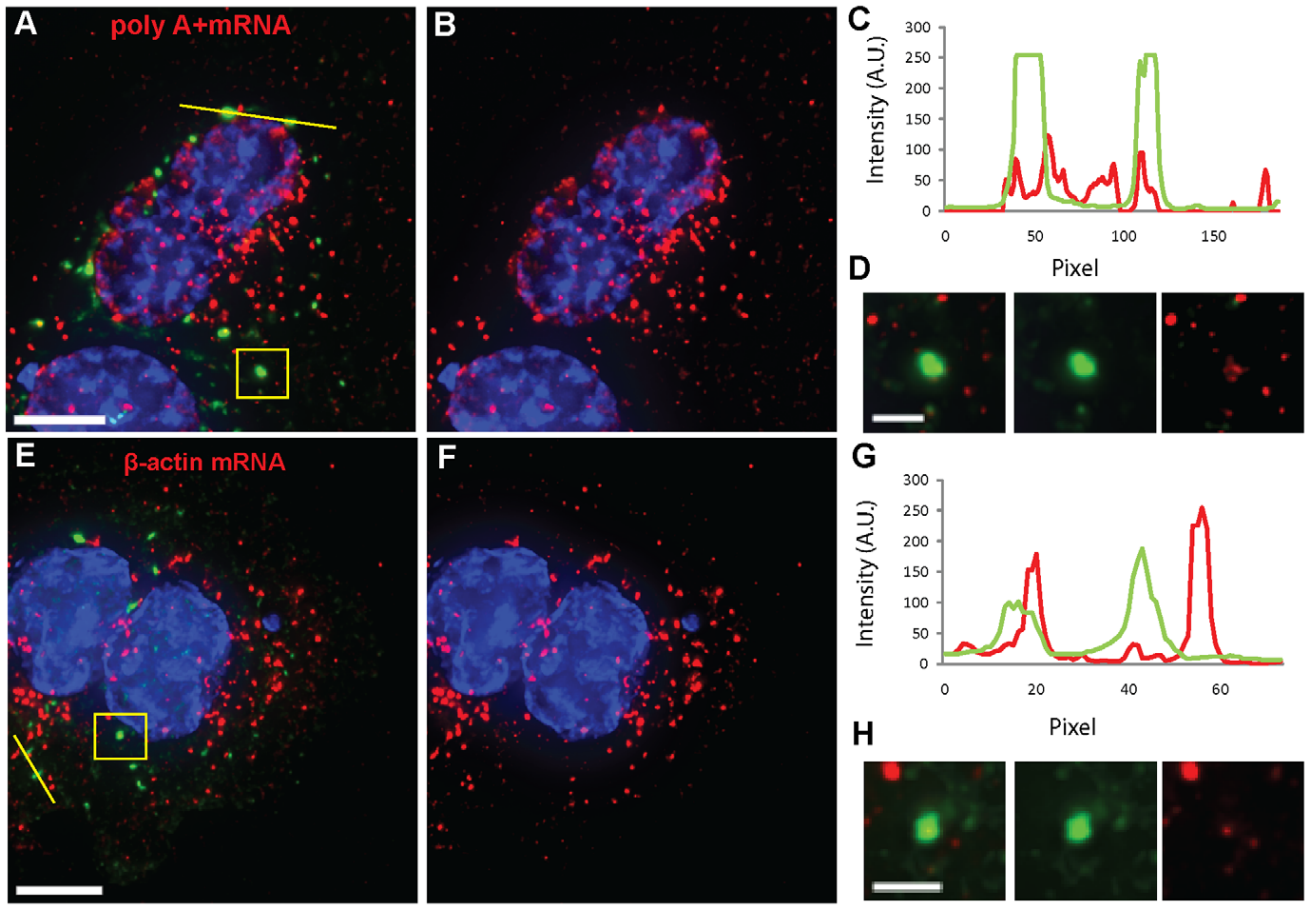
poly A+ and β-actin mRNAs interact with SGs in the presence of Pateamine A. (A, B) Poly A+ and (E, F) β-actin mRNA granules (red) interact with G3BP-stained SGs (green) in U2OS cells as demonstrated by intensity profiles along yellow lines (C, G) and insets displaying magnification of boxed areas (D, H). β-actin mRNA in panel F and in insets was contrast enhanced to allow visualization of granules. Nuclei were stained with DAPI. Scale bars, 10 µm and inset scale bars, 2.5 µm.

After MTRIP delivery, we also treated U2OS cells with 200 nM Pateamine A, an inhibitor of translational initiation that targets eIF4A, a helicase required for the recruitment of ribosomes to mRNAs and causes the formation of stress granules containing TIA-1, eIF4A, eIF4B and G3BP [21]. G3BP-stained SGs formed in all the analyzed cells (Figure 3). Poly A+ mRNA granules interacted with 73% of the analyzed SGs and occupied over 25% of their volume (Figure 3A, B and C); β-actin mRNA granules interacted with 69% of the analyzed SGs and occupied approximately 21% of their volume (Figure 3D, E, F). The difference in recruitment of mRNAs to RNA granules observed in the presence of sodium arsenite and Pateamine A likely depends on the different mechanisms by which these two drugs cause translational inhibition. While the effects of sodium arsenite on translation have extensively been investigated, Pateamine A is a relatively new compound and its mechanism of action has not been completely characterized. A partial characterization of the differences and similarities of SG formation induced by both drugs can be found in Dang *et al.* [22]. SGs formed in the presence of sodium arsenite and Pateamine A share overall a similar protein composition and dynamics of formation. However, Pateamine A induced SGs contain the initiation factor eIF2 (absent in As-induced SGs) and Pateamine A itself. Another interesting feature of the Pateamine A response is that it does not cause an increase in PB formation. This is likely due to the inhibition of nonsense mRNA decay [23]. Overall, this evidence suggests that sodium arsenite and Pateamine A induce mRNA storage/stabilization or decay by different mechanisms.

**Figure 4:**
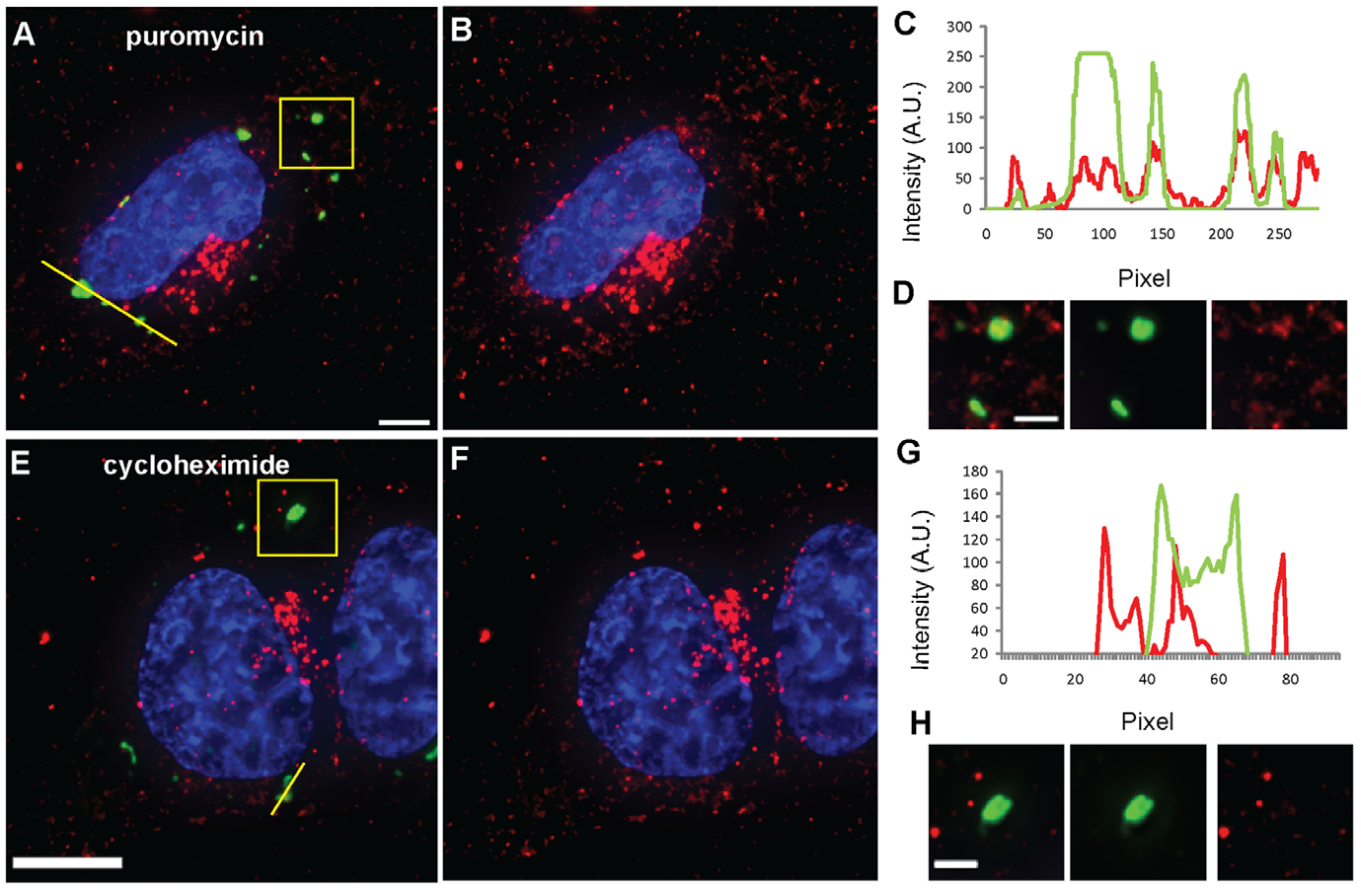
β-actin mRNAs interact with SGs during stress in the presence of puromycin or cycloheximide. β-actin mRNA granules (red) interact with G3BP-stained SGs (green) in U2OS cells during stress in the presence of puromycin (A, B) and cycloheximide (E, F) as demonstrated by intensity profiles along yellow lines (C, G) and insets displaying magnification of boxed areas (D, H). β-actin mRNA in panels B and F and insets was contrast enhanced to allow visualization of granules. Nuclei were stained with DAPI. Scale bars, 10 µm and inset scale bars, 2.5 µm.

In order to ascertain whether MTRIPs allow for the detection of further differences in mRNA recruitment to SGs, we used puromycin and cycloheximide, which cause translational inhibition via two different mechanisms. Puromycin causes premature translational termination by releasing ribosomes from mRNA transcripts and augments SG formation in stressed cells. On the contrary, cycloheximide, which traps mRNAs in polysomes by blocking translational elongation, causes a decrease in the number of sodium arsenite-induced SGs [24]. Despite causing translational inhibition, neither of these drugs was shown to cause SG formation in mammalian cells and yeast [24], [25]. We first analyzed the occupancy of SGs by β-actin mRNAs when translation was inhibited by these two drugs in U2OS cells. As expected, in the presence of puromycin and sodium arsenite all U2OS cells showed large SGs, which stained strongly for G3BP, indicative of high protein concentrations (Figure 4A). In U2OS over 98% of the analyzed SGs were occupied by β-actin mRNA granules, which occupied about 87% of their volume (Figure 4B, C and D). As confirmation, similar experiments were performed using also A549 and DU145 cells, which yielded similar results (Figure S4 and Table 1), showing the versatility of using MTRIPs. No difference, instead, could be observed using MTRIPS targeting poly A+ mRNAs, because, in this case, the SG volume was already fully occupied in the presence of sodium arsenite alone (Table 1). This indicates that probes targeting PolyA+ transcripts are not appropriate for detecting specific changes in mRNA metabolism, as they represent a general population of transcripts.

During stress, cycloheximide treatment dramatically reduced the number of SGs in U2OS, A549 and DU145 cells and those that were still observable in a small number of cells were smaller and stained weakly for G3BP (Figure 4E). Experiments with polyA+ and β-actin mRNA revealed a decrease in SG occupancy in either the percentage of SGs containing mRNA or in the percentage of SG volume occupied by them (Table 1). The differences observed in the analyzed cell lines are likely cell-type specific. Last, we estimated that, on average, 5% and 3% of total poly A+ and β-actin mRNA, respectively, was recruited to SG in the presence of sodium arsenite (Table 2), a percentage in overall agreement with what observed by Mollet *et al.* using both the MS2 tag system and FISH [26].

**Table 2:** Percentage of total mRNAs interacting with SGs and PBs under different experimental conditions.

		Control	As	Puro	As+Puro	Cyclo	As+Cyclo	Noc	As+Noc
% ACTB	inSGs	/	3.1±1.4	/	3.7±1.5	/	1.3±1*	/	4.9±2*
	in PB	0.4±0.1	0.7±0.6	0.6±0.5	0.6±0.5	0.1±0.1	0.6±0.5	0.4±0.4	0.7±0.4
%polyA+	inSGs	/	5±2	/	4.1±1.3	/	1.9±1.3*	/	/
	inPB	0.1±0.1	0.9±0.6*	0.5±0.4	0.5±0.4	0.2±0.2	0.6±0.3	/	/

The percentage of mRNAs interacting with the RNA granules was calculated as described in material and methods in the indicated experimental conditions.

*represents statistically significant difference.

As = sodium arsenite, Pat A = pateamine A, Puro = Puromycin, Cyclo = cycloheximide and Noc = nocodazole.

**Figure 5:**
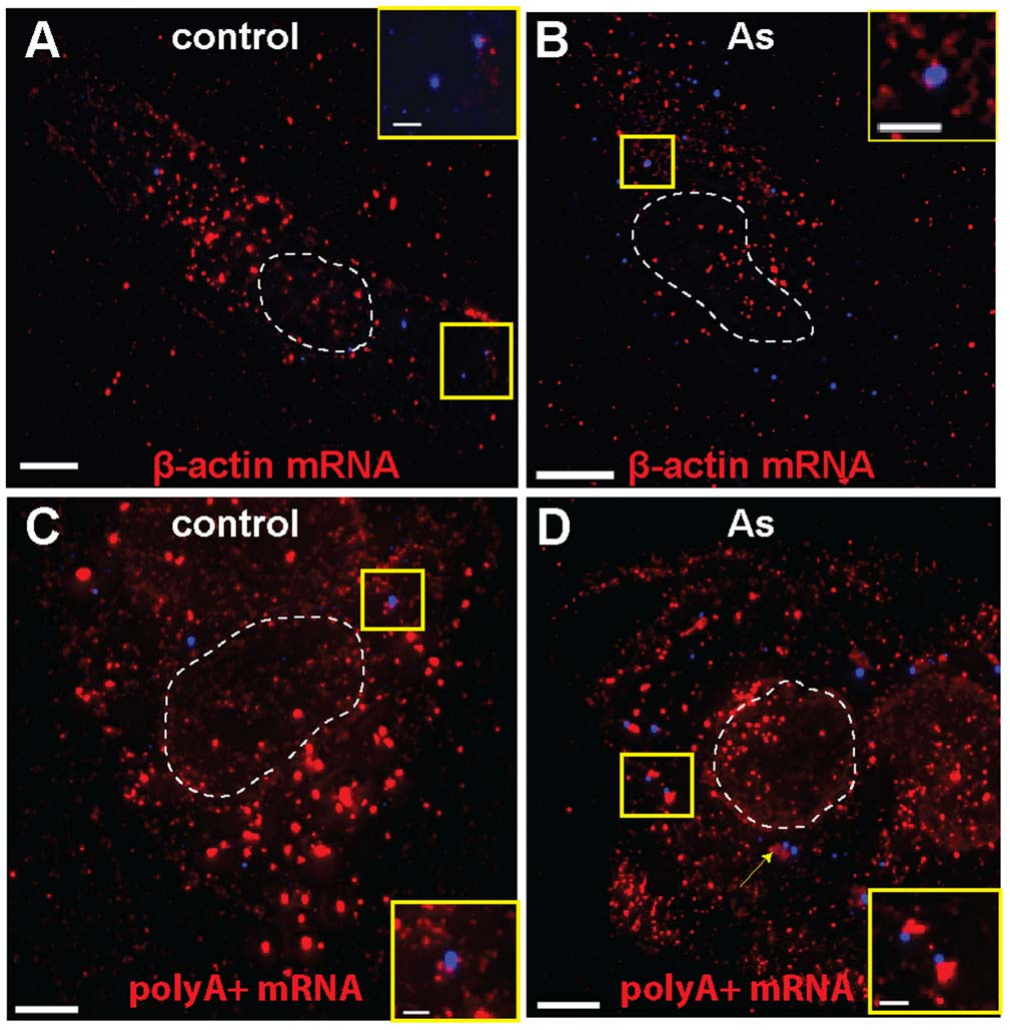
β-actin and poly A+ mRNA interactions with PBs. Dcp1α-stained PBs (blue) interact with β-actin (A and B) and poly A+ (C and D) mRNA granules (red) in untreated U2OS cells and in the presence of sodium arsenite as demonstrated by insets displaying magnification of boxed areas. β-actin mRNA in insets was contrast enhanced to allow visualization of granules. The arrow in panel D indicates a SG-localized mRNA interacting with one PB. Dashed lines indicate the position of nuclei. Scale bars, 10 µm and inset scale bars, 2.5 µm.

We used a similar approach to investigate mRNA interactions with PBs, which are considered sites of mRNA degradation. Under normal growth conditions, SLO exposure did not alter PB number, while, following sodium arsenite exposure, a small decrease (25%) in PB number was observed (Figure S3D). We delivered the MTRIPs targeting β-actin mRNAs into live cells, and subsequently immunostained for DCP1a after fixation. Under typical growth conditions U2OS cells contained few PBs, approximately 48% of which interacted with mRNA granules (Figure 5A). Upon sodium arsenite treatment for 1 hour the number of PBs per cell increased, as expected, and 72% of them were found to interact with β-actin mRNAs (Figure 5B). Such interactions further increased during stress in the presence of puromycin while they decreased in the presence of cycloheximide (data not shown and Table 3). We also analyzed PB interactions with poly A+ mRNAs (Figures 5C and D and Table 3). Note that in the polyA+ case, the large number of mRNA granules recruited to the SGs makes it possible to approximate the SG location and observe interactions with PBs (Figure 5D).

**Table 3:** PB occupancy by mRNAs in different experimental conditions.

		Control	As	Puro	As+Puro	Cyclo	As+Cyclo	Noc	As+Noc
ACTB	%PBs	48	72	60	87	62	59	43	43
	% PB vol	62±32	41±19	22±19*	41±14	28±20	43±20	27±22	34±26
polyA+	%PBs	48	51	62	42	38	43	/	/
	% PB vol	17±14	59±41	48±32	42±21	59±37	46±32	/	/

Percentage of PBs (% PBs) and percentage of PB volume (% PB vol) occupied by mRNA granules in all the analyzed experimental conditions.

*represents statistically significant difference.

As = sodium arsenite, Pat A = pateamine A, Puro = Puromycin, Cyclo = cycloheximide and Noc = nocodazole.

In addition, the representative cells in Figure 5 clearly show that most mRNA granules are larger than a PB, which is approximately the size of our microscope objective's point-spread-function, ∼250 nm. Therefore, even though we cannot directly assess PB function, our data indicate that native mRNAs do not likely accumulate in PBs but rather interact with them. Last, we estimated that less than 1% of the total mRNA (both β-actin and poly A+) interacted with PBs, only partially occupying their volume independently on the experimental condition (Table 2). This measurement is in overall agreement with the percentage determined by Franks *et al.* using plasmid derived mRNA [27] and by Stohr and colleagues who rarely observed endogenous mRNAs in PBs, which led them to suggest that during stress, mRNAs shuttled to PBs were rapidly decayed and therefore could not be detected [13].

### mRNA changes its localization during translational initiation inhibition

**Figure 6:**
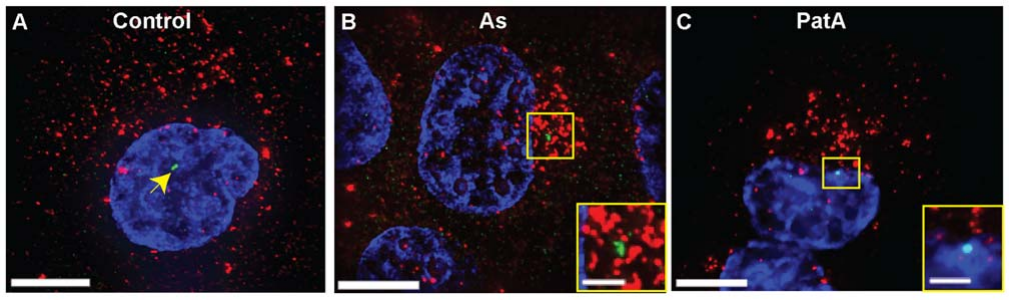
β-actin mRNAs are recruited to the MTOC upon treatment with sodium arsenite and Pateamine A. β-actin mRNA granules (red) are distributed in the cytoplasm in untreated U2OS cells (A) and localize near the MTOC (stained with a γ-tubulin antibody, green) after treatment with sodium arsenite (As) (B) and Pateamine A (PatA) (C), but no colocalization is observed as indicated in the figures insets. In panel A the MTOC is indicated by the arrowhead. Nuclei were stained with DAPI. Scale bars, 10 µm and inset scale bars, 2.5 µm.

**Figure 7 pone-0019727-g007:**
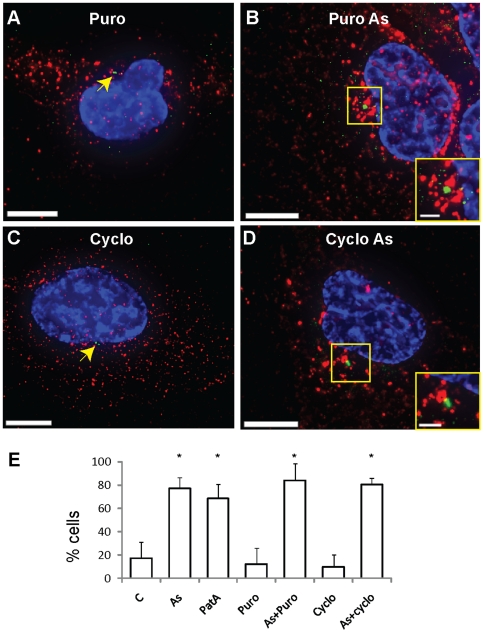
β-actin mRNAs are recruited to the MTOC during stress in the presence of puromycin or cycloheximide. β-actin mRNA granules (red) remain distributed in the cytoplasm of U2OS cells treated with puromycin (Puro) (A) or cycloheximide (Cyclo) (C), while localize near the MTOC (stained with a γ-tubulin antibody, green) when sodium arsenite (As) is also added (B and D). No colocalization between mRNAs and the MTOC is observed as indicated in the figures insets. In panels A and C the MTOC is indicated by the arrowhead. Nuclei were stained with DAPI. Scale bars, 10 µm and inset scale bars, 2.5 µm. (E) Percentage of cells that show recruitment of mRNAs to the MTOC (% cells) in the indicated experimental conditions; error bars indicate standard deviation. * represents statistically significant difference (P<0.05).

The comparison between the representative cells in Figures 6A, B and C clearly shows that a portion of mRNAs, in the presence of both sodium arsenite and Pateamine A, dramatically changed their localization and moved near the nucleus, where they appeared to be larger and brighter compared with the granules distributed within the cytoplasm and in protrusions. By staining for γ-tubulin, we verified, first of all, that these mRNAs localized near the microtubule organizing center (MTOC) (Figure 6A, B, and C), even though precise colocalization between the MTOC and β-actin mRNAs was not observed (insets in Figures 6 B and C). We observed a similar change in β-actin mRNA localization in U2OS cells upon treatment with sodium arsenate at 2.5 mM for 1 h at 37°C and with 1 mM and 2 mM sodium arsenite for 1 hour at 37°C (data not shown). In the latter case, recruitment of mRNA granules to the region near the MTOC was more evident because, approaching lethal concentrations, the cells were smaller and rounded (data not shown). Moreover, we obtained similar results using Poly A+-targeting probes or β-actin mRNA probes in A549 cells, indicating that this was neither an mRNA-specific, nor a cell-specific response (Figure S5A to D) and during stress, in the presence of puromycin and cycloheximide (Figure 7A–D). The fact that puromycin and cycloheximide alone did not induce such mRNA re-localization indicates that this process, exactly like SG formation, is a consequence of translational initiation inhibition and does not depend specifically on eIF2α phosphorylation. By visual inspection, we estimated that over 80% of U2OS showed an obvious localization of mRNAs near the MTOC in the presence of sodium arsenite, with or without puromycin or cycloheximide versus ∼17% of the cells under various control conditions. Similarly, 200 nM Pateamine A affected 68% of the cells (Figure 7E).

**Figure 8:**
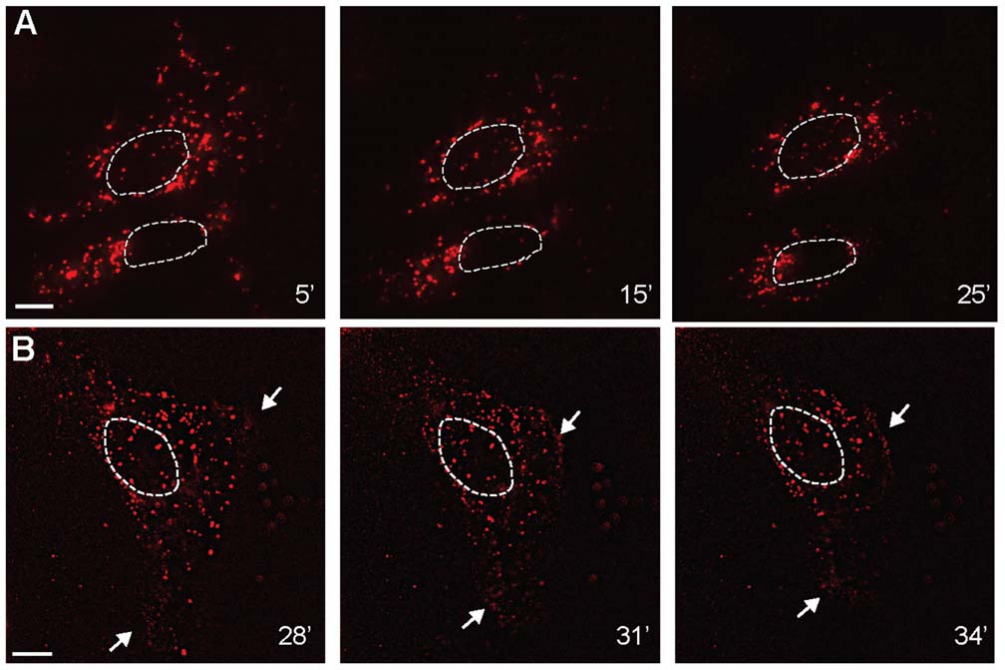
Observation of β-actin mRNA recruitment to the MTOC using live cell imaging. (A) Time-lapse imaging of a U2OS cell showing recruitment of β-actin mRNA to the MTOC using widefield deconvolution imaging and (B) single optical plane showing morphological changes induced by sodium arsenite exposure. Arrows indicate retraction of cell edges, nuclei position is indicated by dotted line. All times, in minutes, indicate time after sodium arsenite exposure.

We subsequently tested MTRIPs' ability to visualize the motion of single β-actin mRNA granules toward the MTOC during the stress response via live cell imaging. We delivered 6 MTRIPs (15 nM each, Table S1), 4 designed to target the β-actin mRNA coding region and 2 designed to target the 3′UTR region. We did so in order to increase i) the signal-to-background ratio and ii) the observation time by increasing the number of fluorophores per RNA (unpublished results). Both in the absence and presence of sodium arsenite, the mRNA granules in the cytoplasm exhibited dynamic behavior, with granules interacting transiently, undergoing anterograde as well as retrograde motion and pauses. Granules along the cell edges and perinuclear region were instead mainly static, probably as a result of anchoring to the complex cytoskeletal network in these cellular compartments (Movie S1). In order to observe the overall changes in mRNA distribution within the cell cytoplasm, we collected z-stacks every 5 min (Figure 8A), and each image was subsequently deconvolved to remove out of focus light. The analysis revealed that, approximately 15 minutes after sodium arsenite addition, the β-actin mRNA granules present in the cytoplasm started to accumulate near the nucleus and, in most cases, after 30 minutes, the cells underwent dramatic changes in their morphology, because their edges proceeded to retract toward the nucleus (Figure 8B). Sodium arsenite exposure has been shown to cause alterations in cell adhesion to the cell culture substrate at concentrations well below the concentration used here, as well as alterations in cell migration and focal adhesion localization [28]. Interestingly, this effect seemed to facilitate, at least in part, the migration of β-actin mRNA granules toward the nucleus. Live cell imaging of a single optical plane at 1 Hz over approximately 10 min revealed that such morphological changes occurred progressively over ∼5 minutes and indicated that the mRNA granules at the cell periphery were not recruited toward the MTOC, possibly because they remain anchored to the cytoskeleton (Figure 8B).

### MTRIPS do not affect mRNA interactions with SGs and PBs and localization near the MTOC

In order to demonstrate that mRNA targeting by our probes unlikely affect their metabolism, we repeated the experiments using traditional fluorescence in situ hybridization (FISH) combined with immunostaining. Once the optimal probe concentration and hybridization conditions were optimized to reduce the background signal using untargeted probes (Figure S6 A and B), we performed FISH and stained with TIAR or DCP1, after rinsing abundantly with phosphate buffered saline to remove excess formamide. The resulting staining was similar to traditional immunostaining for both proteins, with TIAR prevailing in the nucleus in control experiments (Figure S6C) and in SGs upon sodium arsenite exposure (Figure S6D), and with DCP1a aggregating in PBs (Figures S6E and F). FISH confirmed the results obtained by live cell delivery using MTRIPs. β-actin mRNA granules were distributed through the cells in control experiments (Figure S6B,C and E) while, in the presence of sodium arsenite, single mRNA granules interacted with SGs and PBs (Figures S6D and F). We also confirmed that a portion of β-actin mRNA migrated toward the nucleus (Figure S6D and F, arrows), even if the hybridization conditions used did not allow us to stain successfully for γ-tubulin. FISH results confirmed that the mRNA granules along the edges of the cell remained attached to the cytoskeleton in agreement with the results obtained by live cell imaging (Figure S6D, arrow).

### Microtubule integrity is necessary for β-actin mRNA localization near the MTOC and for interactions with SGs and PBs

**Figure 9:**
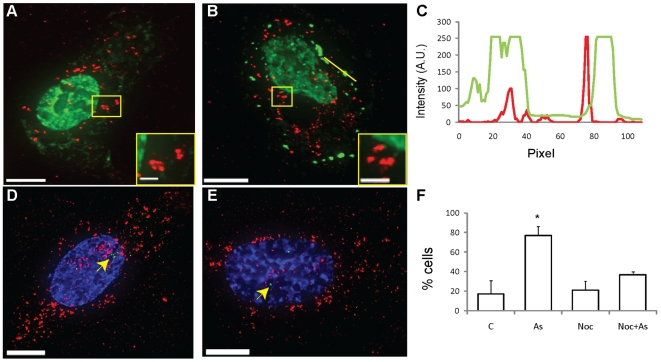
Effect of nocodazole on β-actin mRNA interaction with SGs and PBs and localization to the MTOC. Distribution of β-actin mRNAs (red) in U2OS cells and TIAR staining (green) after nocodazole treatment (A) without and (B) with sodium arsenite and interaction with TIAR-stained SGs as demonstrated by intensity profile along yellow line (C). Insets displaying magnification of boxed areas show clusters of mRNA granules in the cytoplasm. The mRNA granules do not localize near the MTOC (green and indicated with arrow) in the presence of nocodazole without (D) or with (E) sodium arsenite. (F) Percentage of cells that show recruitment of mRNAs to the MTOC (% cells) in the indicated experimental conditions (C = control, As = sodium arsenite and Noc = nocodazole); error bars indicate standard deviation,* represents statistically significant difference (P<0.05).

In the previous paragraphs we demonstrated that MTRIPs targeting specifically β-actin mRNA are extremely sensitive in detecting small changes in the interactions between transcripts and SGs or PBs. Therefore, we also used these probes to test whether such interactions depend on an intact microtubule network. Microtubule disruption has been shown to induce dramatic alterations in both the assembly and the spatial localization of SGs and PBs in different cell lines [29], [30], [31]. We investigated the effect of nocodazole, a well known microtubule disrupting agent, on β-actin mRNA localization to SGs and PBs as well as to the MTOC during stress. First, by staining with either an antibody against α-tubulin or with phalloidin, we observed that, in unstressed cells, β-actin mRNAs are distributed throughout the cytoplasm, interacting with both microtubules and stress fibers (Figure S7 A to D). Second, by staining with an antibody against α-tubulin, we demonstrated that the mRNAs that localize near the MTOC as a consequence of sodium arsenite treatment colocalized with microtubules (Figure S7 E and F). Third, we induced microtubule disruption in U2OS cells after performing a nocodazole titration up to 20 µM; we chose concentrations low enough to avoid complete cell withdrawal and cell rounding, while allowing preservation of cell morphology (data not shown). Microtubule disruption in untreated cells did not alter the staining for endogenous proteins such as TIAR, which maintained a prevalent nuclear staining (Figure 9A). Instead, the distribution of β-actin mRNA granules within the cells was altered, since single granules appeared to form “clusters” distributed within the cell cytoplasm (Figure 9A inset). Experiments with nocodazole and sodium arsenite were performed by incubating the cell with nocodazole for 30 minutes at 37°C and then with 0.5 mM arsenite and nocodazole for an additional hour. Approximately 60% of the U2OS cells formed SGs, which appeared to be small, more evenly dispersed within the cell cytoplasm instead of being predominately perinuclear and colocalized with microtubules (Figure 9B and S7 G and H). Approximately 79% of the analyzed SGs contained or interacted with mRNA granules, which occupied about 34% of their volume (Figure 9B and Table 1). We obtained similar results using nocodazole after targeting β-actin mRNAs in A549 cells (Figure S7I and J and Table 1). Microtubule disruption not only impaired significantly the interactions between mRNAs and SGs but also with PBs, as reported in Table 3 (and data not shown), and greatly reduced mRNA localization near the MTOC (Figures 9 D, E and F). Indeed, in this experimental condition, the mRNAs tended to remain distributed in clusters in the cytoplasm (Figure 9B inset). We observed a similar distribution of both mRNA granules and SGs using vinblastin, another microtubule disrupting agent (Figure S7 K and L). These results demonstrate that mRNA localization near the MTOC and efficient interactions with SGs and PBs are dependent on an intact microtubule network

### β-actin mRNAs near the MTOC interact with RNA exosome granules during the stress response

Above we demonstrated that stress caused mRNAs to re-localize within the cell: some remain attached to the cytoskeleton at the cell edges, some interact with SGs and PBs in the cell body and some localize near the MTOC. Once established that mRNA localization near the MTOC occurred independently of eIF2α phosphorylation and in a microtubule dependent manner, we investigated the role of this cellular region during stress. All the mRNA binding proteins known to be involved in the stress response such as HuR, TTP, BRF-1 TIAR/TIA-1, etc. have been mainly analyzed as a consequence of their localization to SGs and PBs and, generally, they were found to have roles in mRNA transcription, translation, silencing, decay and stability [4]. Similarly, ZBP1, which has a key role in mRNA stabilization, was shown to localize to SGs upon sodium arsenite treatment. While no relationship between the MTOC and SGs has ever been observed [17], PB localization near the centrosome has been described by Aizer *et al.* [32], suggesting an intriguing role for the observed mRNA localization to this cellular region. However, the vast majority (over 70%) of the cells that we analyzed showed no localization of either SGs or PBs near the the MTOC (Figure S8).

**Figure 10:**
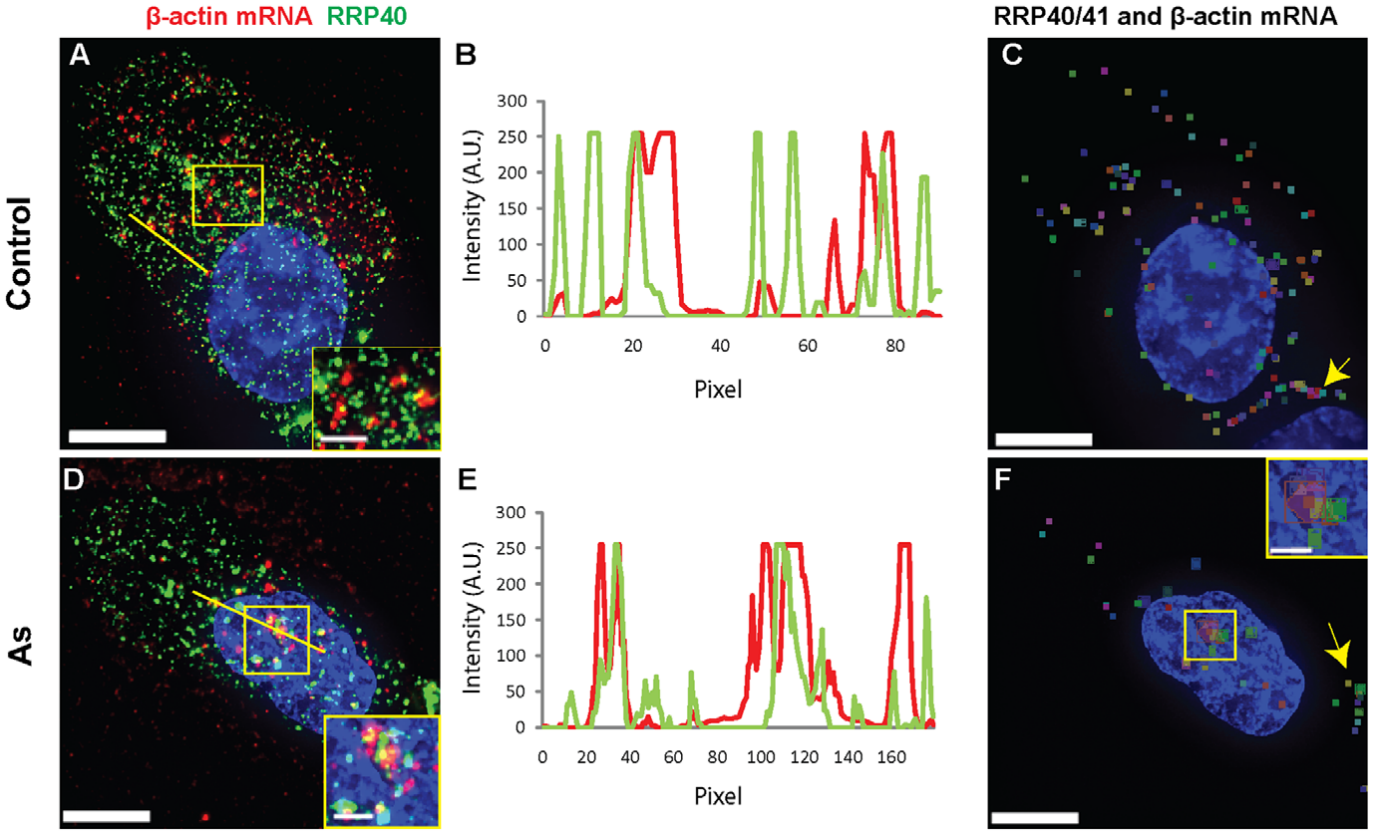
β-actin mRNAs colocalize with exosome subunits-enriched granules near the nucleus during stress. RRP40 colocalizes with β-actin mRNAs in control cells (A) and in cells treated with sodium arsenite (D) as demonstrated by insets displaying magnification of boxed areas and intensity profiles along yellow lines (B and E). Results of the analysis performed in Volocity showing the cytoplasmic distribution of colocalized RRP40/41 and mRNA granules (colored squares) in control cells (C) and in the presence of sodium arsenite (F). Nuclei were stained with DAPI. Scale bars, 10 µm and inset scale bars, 2.5 µm.

Given that cytoplasmic granules containing exosomal subunits described by Lin *et al.* [18], localized near the nucleus and did not correspond to either SGs or PB, we proceeded to investigate the localization of the 3′-to-5′ decay machinery during the stress response. First, we analyzed the distribution within the cytoplasm of two previously characterized exosome subunits, RRP40 and RRP41 and verified that their overall localization did not change in the presence of sodium arsenite, independent on SLO treatment. In both unstressed and stressed cells, they colocalized along the cell edges and showed cytoplasmic staining characterized, as described, by both small granules and well defined foci present in the cytoplasm of some cells (Figure S9 A–D) [18]. Only exosome granules containing both RRP40 and RRP41 were used to demonstrate colocalization with MTRIPs targeting β-actin mRNA by means of intensity profiles (Figure 10A, B, D and E) and as described in the Material and Methods section. In over 75% of the analyzed control cells, interactions between mRNAs and the exosome subunits enriched in RRP40 and RRP41 occurred both along the cell edges and in granules dispersed in the cytoplasm (Figures 10 C). In the presence of sodium arsenite, such interactions prevailed not only along the cell edges but also near the nucleus (in ∼65% of the cells), where the mRNAs localized as a result of translational inhibition (Figures 10 F). Overall the data indicates that sodium arsenite induced mRNAs to localize near the MTOC, likely to interact with the 3′to5′ decay machinery for degradation.

## Discussion

Utilizing live cell hybridization assays combined with immunofluorescence, we have demonstrated how multiply labeled tetravalent imaging probes (MTRIPs) can be used to characterize with single RNA granule sensitivity the localization of native poly A+ and β-actin mRNAs during translational inhibition. Our experiments were performed in different epithelial cell lines and different experimental conditions, demonstrating the versatility of our probes.

The use of MTRIPs permitted, first of all, the quantification of the colocalization of native mRNA with SG and PB-associated proteins and the observation of the changes induced by different treatments such as sodium arsenite (Figure 2 and 5), that triggers eIF2α phosphorylation, Pateamine A (Figure 3), which targets eIF4A, and in the presence of puromycin and cycloheximide (Figure 4), which further alter mRNA translational potential. Our results were overall consistent with those obtained using the MS2 tag system [26], [27]. Plasmid derived system, however, express transcripts often lacking native UTRs or complete sets of introns, possibly leading to aberrant localization and regulation. Moreover, they might suffer from overexpression, which may lead to conclusions that do not represent the behavior of native molecules, usually expressed at much lower levels [31]–[32]. On the contrary, here we aimed to perform our experiments respecting the physiological stoichiometry of protein/mRNA interactions by monitoring the localization of native RNAs and protein. Last, our results, supported by traditional FISH analysis, also strongly indicate that MTRIPs unlikely affected mRNA capability to be properly processed and we feel that this will be a critical strategy for further evaluating the working model of cytoplasmic mRNA metabolism, the “mRNA lifecycle” [5].

Prior to this investigation, SGs and PBs were the only RNA granules extensively described to be involved in the stabilization or degradation of transcripts during stress and the identification of putative proteins involved in the stress response was based on their localization to these regulatory foci. While our results are consistent with previous work, that indeed SGs and PBs represent sites where mRNAs are recruited upon release from polysomes as a consequence of translational silencing, the small percentage of mRNA involved in these transactions suggests that mRNA stabilization and degradation during stress may not rely exclusively on recruitment to these foci. Indeed, recently, has been proposed that ER-bound transcripts escape interactions with SGs during stress [14]; in yeast, mutations that prevented SG formation did not affect the stabilization of mRNAs, which also imply the existence of alternative factors [33]. According to this hypothesis, we observed here for the first time in epithelial cells that some mRNAs remain attached to the cytoskeleton at the cell edges and some localize near the MTOC (Figures 6–8), where they clearly interact with granules or foci enriched in exosome subunits (Figure 10). Unfortunately, in this case, accurate quantification in the perinuclear region was complicated by the close spacing and observed aggregation of mRNA granules.

Although we have not yet demonstrated mRNA degradation in these granules, our observations suggest how the 3′ to 5′ decay machinery may participate in the stress response. Moreover, our evidence of interactions between mRNAs and exosome components is in agreement with recent work that suggested that not only the 5′ to 3′ decay machinery (PBs) but also the 3′ to 5′ are involved in the decay of unstable ARE-containing mRNAs in mammalian cells [34]. These two degradation pathways are indeed known to be redundant and not mutually exclusive and therefore it would not be surprising if they were also restricted to the same cellular compartment rather than spatially segregated [34].

Last, using MTRIPs, we demonstrated that both mRNA localization near the MTOC and efficient interactions between SGs and PBs are impaired by the addition of nocodazole during stress (Figure 9). Therefore, not only is an intact network critical for PB and SG formation and dynamics, as previously shown [29], [30], [31], but also for proper mRNA processing. We feel that this observation supports and reemphasizes the role of microtubules in mRNA transport in the cytoplasm, especially during stress, when it has not been analyzed in detail. Several hypotheses have been proposed regarding mRNA transport, such as transport on microtubules, or on microfilaments, or via diffusion throughout the cytoplasm, controlled by the size of the mRNA and not by its sequence [35], [36], [37], [38], [39], [40], [41]. Our results imply that diffusion alone does not represent an efficient mechanism for mRNA transport and that microtubule integrity is required for efficient localization of mRNAs to putative sites of translational regulation.

In conclusion, MTRIPs allowed for efficient labeling in both live and fixed cells of native mRNA to gain quantitative and qualitative information regarding interactions with SGs, PBs and exosome granules. We feel that MTRIPs represent a powerful tool for the future study on RNA dynamics and will be essential to examine the specific roles of AU-rich-specific cis and trans-acting factors on mRNA movement during translational repression. Future work will also focus on exploring the combination of MTRIPs and subdiffraction-limited imaging techniques to more accurately quantify mRNA /RNA granule interactions.

## Supporting Information

Figure S1 **Characterization of MTRIPs targeting β-actin mRNA.** Single plane images showing U2OS cells treated with SLO without (A) or with (B) MTRIPS targeting β-actin mRNA, imaged at the same exposure time (126 ms) and with similar contrast enhancement. In order to test MTRIPs specificity, MTRIPS targeting β-actin mRNA (C, red) or the genomic RSV RNA (D, green) were delivered at the same concentration (30 nM). The merged image in E demonstrates no colocalization between targeted and “scrambled” probes. Nuclei were stained with DAPI. Scale bars, 10 µm.
(TIF)

Figure S2 **MTRIPs do not affect target mRNA stability and translatability.** (A) mRNA decay in cells treated with SLO without or with MTRIPs was assayed upon treatment with Actynomicin D after 0, 4, 8 and 24 h as described in the text via qRT-PCR. β-actin mRNA expression fold change is normalized to GAPDH. (B) Percentage of cells expressing GFP-β-actin in control cells and in the presence of 50 nM or 200 nM siRNA or 30 nM MTRIPs. Error bars indicate standard deviation and * represents statistically significant difference (P<0.05).
(TIF)

Figure S3 **SLO treatment does not alter SG/PB formation and/or protein composition.** Untreated U2OS cells (A) or treated with SLO (B) formed SGs that contain endogenous HuR, G3BP and TIAR proteins after treatment with 0.5 mM sodium arsenite for 1 h at 37°C. Scale bars, 10 µm. (C) Average number of SGs per cell observed upon sodium arsenite treatment with and without SLO. (D) Average number of PBs per cell in untreated (-) and treated cells (As) with and without SLO. Error bars indicate standard deviation and * represents statistically significant difference (P<0.05).
(TIF)

Figure S4 **β-actin mRNAs interact with SGs during the stress response in A549 and DU145 cells.** β-actin mRNA granules (red) interacted with G3BP or TIAR-stained SGs (green) in A549 (A) and DU154 cells (D) as demonstrated by intensity profiles along yellow lines (B and E) and insets displaying magnification of boxed areas (C and F). Nuclei were stained with DAPI. Scale bars, 10 µm and inset scale bars, 2.5 µm.
(TIF)

Figure S5 **mRNAs localization near MTOC is a general mechanism during the stress response.** Poly A+ (A) and β-actin (C) mRNA granules (red) are distributed in the cytoplasm in untreated U2OS cells and A549 cells, and localize near the MTOC (stained with a γ-tubulin antibody, green) after treatment with sodium arsenite (As) (B and D). Such mRNA localization was observed in 80% of U2OS and 66% of A549 respectively (data not shown). No colocalization between mRNAs and the MTOC is observed as indicated in the figures insets. In panels A and C the MTOC is indicated by the arrowhead. Nuclei were stained with DAPI. Scale bars, 10 µm and inset scale bars, 2.5 µm.
(TIF)

Figure S6 **Specific detection of β-actin mRNA using FISH and interactions with SGs and PBs.** Cells were hybridized with scrambled probes (A) or with linear probes targeting β-actin mRNA (B) as described in Material and Methods using similar exposure times (303 ms) and contrast enhancement. β-actin mRNA distribution in U2OS without (C and E) and with (D and F) sodium arsenite observed using FISH and immunofluorescence. Interactions with SGs and PBs are demonstrated by insets displaying magnification of boxed areas. β actin mRNAs along the cell edge and near the nucleus are indicated by the arrows. Scale bars, 10 µm and inset scale bars, 2.5 µm. Nuclei were stained with DAPI.
(TIF)

Figure S7 **β-actin mRNA interaction with microtubules and stress fibers and effect of microtubule disruption.** In unstressed U2OS cells, β-actin mRNA (ACTB) granules (red) colocalized with microtubules (MT) (A) and stress fibers (C) as shown by insets displaying magnification of boxed areas and profiles along yellow lines (B and D). (E and F) Upon sodium arsenite (As) treatment, β-actin mRNA granules colocalization with α-tubulin stained microtubules near the nucleus increases, as shown by inset displaying magnification of boxed area. (G and H) Treatment with nocodazole (Noc) disrupted microtubules (green) and impaired formation of arsenite mediated SG (red). Effect of nocodazole in A549 cells (I), or of vinblastin (Vin) in U2OS cells (K) in the presence of sodium arsenite. mRNAs (red) remained distributed in the cytoplasm, where they formed clusters (insets), and interacted with small SGs (blue), as seen by the profiles along yellow lines (J and L). Disruption of microtubules was demonstrated by staining for α-tubulin (green). Nuclei were stained with DAPI. Scale bars, 10 µm and inset scale bars, 2.5 µm.
(TIF)

Figure S8 **SGs and PBs do not localize near the MTOC during the stress response.** G3BP-stained SGs (A) and DCP1-stained PBs (B) do not colocalize with the mRNAs near the MTOC. In (A) the MTOC was stained with a γ-tubulin antibody (green) and indicated by the arrow while in (B) the position of the MTOC, indicated by the arrow, was assessed by staining with an α-tubulin antibody (inset). Nuclei position is indicated by dotted line. Scale bars, 10 µm.
(TIF)

Figure S9 **Distribution of exosome subunits-enriched granules in the cytoplasm of U2OS cells.** Colocalization of RRP40 and RRP41 in untreated cells (A and B) and treated with sodium arsenite (C and D) along cells edges (arrows) and in cytoplasmic granules as demonstrated by insets displaying magnification of boxed areas. The overall distribution of the granules is not altered by SLO treatment. Nuclei were stained with DAPI. Scale bars, 10 µm and inset scale bars, 2.5 µm.
(TIF)

Table S1 Sequences of MTRIPs targeting â-actin mRNA (ACTB) and the polyA tail with location within transcript.
(DOC)

Table S2 SGs occupancy by â-actin mRNA in U2OS cells treated with increasing sodium arsenite concentrations.
(DOC)

Movie S1 Motion of β-actin mRNA granules (red) in a U2OS cell after exposure to sodium arsenite. Cells were imaged at 37°C and at 1 timepoint per sec.
(MOV)
