# Supplementary figures and images for: Characterizing mRNA Interactions with RNA Granules during Translation Initiation Inhibition
Source: PLoS One. 2011 May 5;6(5):e19727. doi: 10.1371/journal.pone.0019727 (PMC3088712; doi:10.1371/journal.pone.0019727)

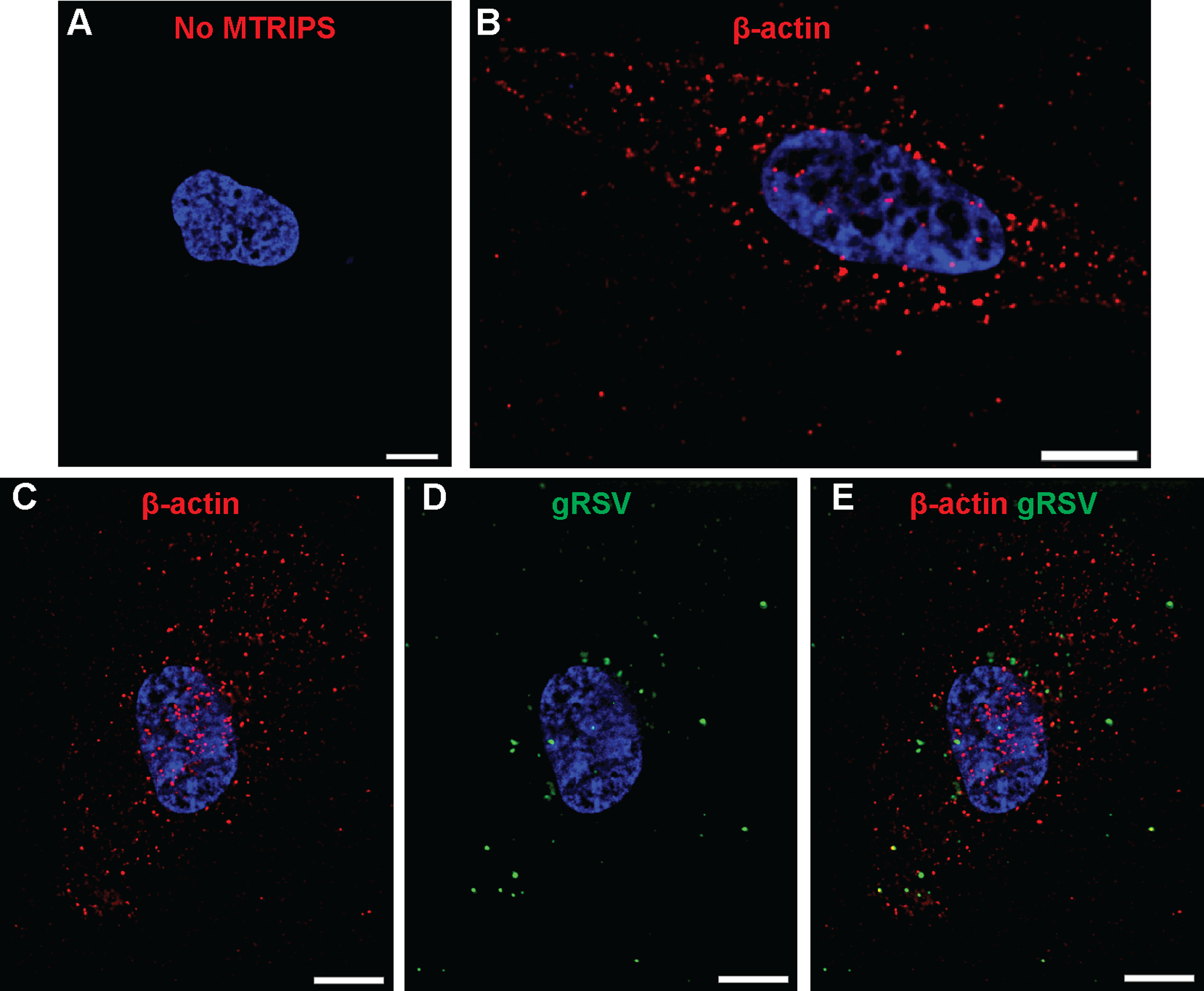

Supplement: Figure S1 — Characterization of MTRIPs targeting β-actin mRNA. Single plane images showing U2OS cells treated with SLO without (A) or with (B) MTRIPS targeting β-actin mRNA, imaged at the same exposure time (126 ms) and with similar contrast enhancement. In order to test MTRIPs specificity, MTRIPS targeting β-actin mRNA (C, red) or the genomic RSV RNA (D, green) were delivered at the same concentration (30 nM). The merged image in E demonstrates no colocalization between targeted and “scrambled” probes. Nuclei were stained with DAPI. Scale bars, 10 µm. (TIF) [file pone.0019727.s001.tif]

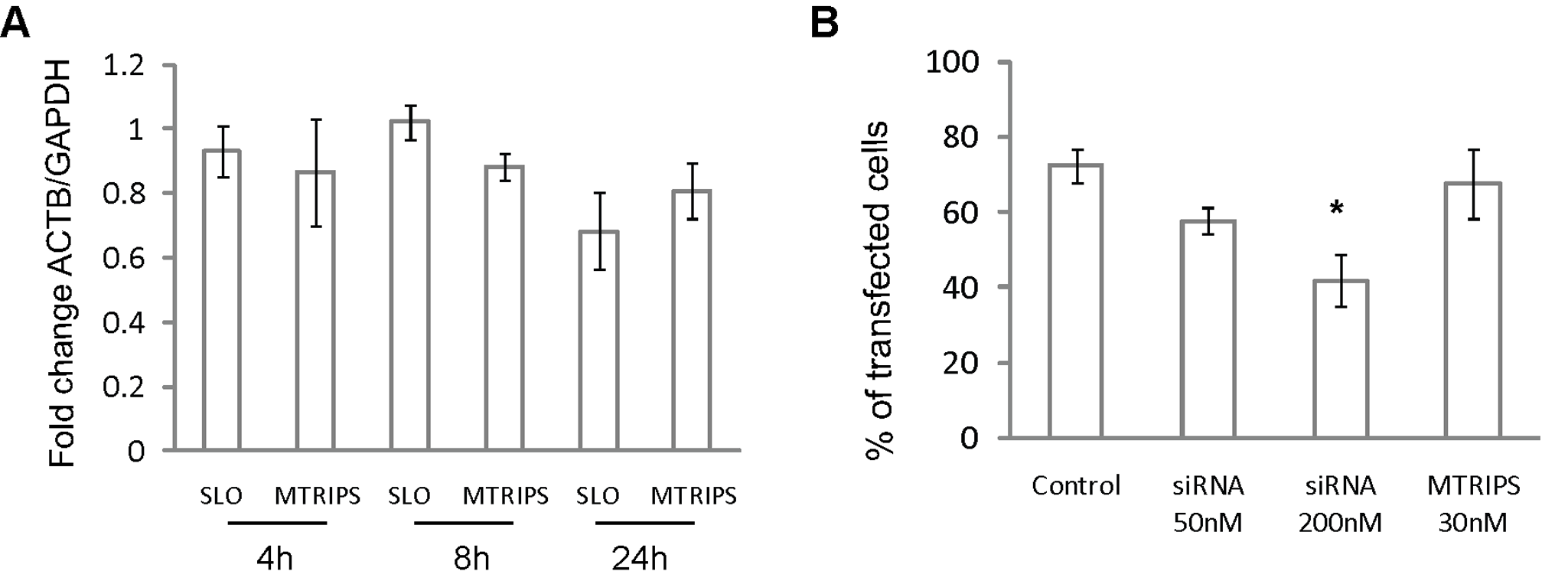

Supplement: Figure S2 — MTRIPs do not affect target mRNA stability and translatability. (A) mRNA decay in cells treated with SLO without or with MTRIPs was assayed upon treatment with Actynomicin D after 0, 4, 8 and 24 h as described in the text via qRT-PCR. β-actin mRNA expression fold change is normalized to GAPDH. (B) Percentage of cells expressing GFP-β-actin in control cells and in the presence of 50 nM or 200 nM siRNA or 30 nM MTRIPs. Error bars indicate standard deviation and * represents statistically significant difference (P<0.05). (TIF) [file pone.0019727.s002.tif]

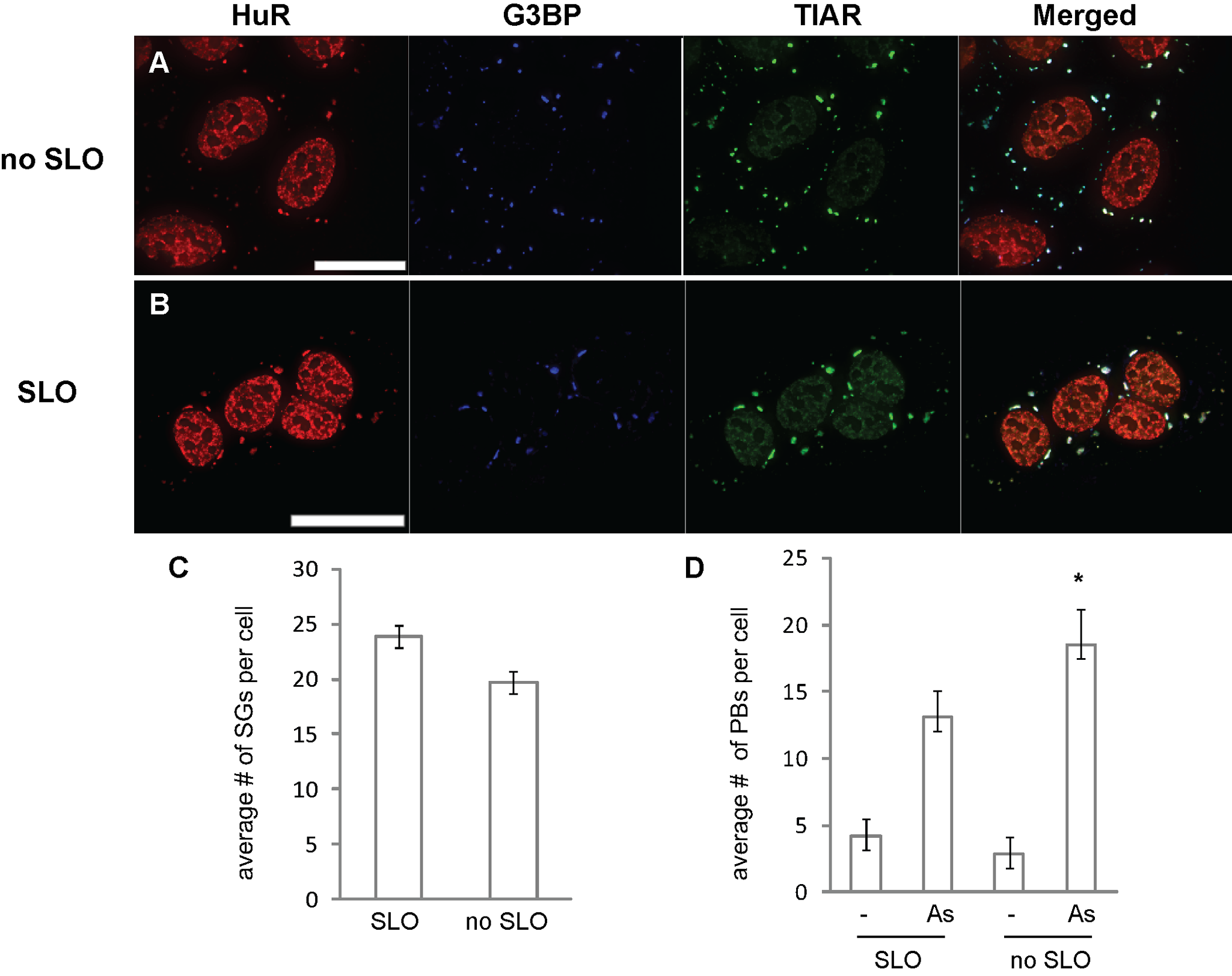

Supplement: Figure S3 — SLO treatment does not alter SG/PB formation and/or protein composition. Untreated U2OS cells (A) or treated with SLO (B) formed SGs that contain endogenous HuR, G3BP and TIAR proteins after treatment with 0.5 mM sodium arsenite for 1 h at 37°C. Scale bars, 10 µm. (C) Average number of SGs per cell observed upon sodium arsenite treatment with and without SLO. (D) Average number of PBs per cell in untreated (-) and treated cells (As) with and without SLO. Error bars indicate standard deviation and * represents statistically significant difference (P<0.05). (TIF) [file pone.0019727.s003.tif]

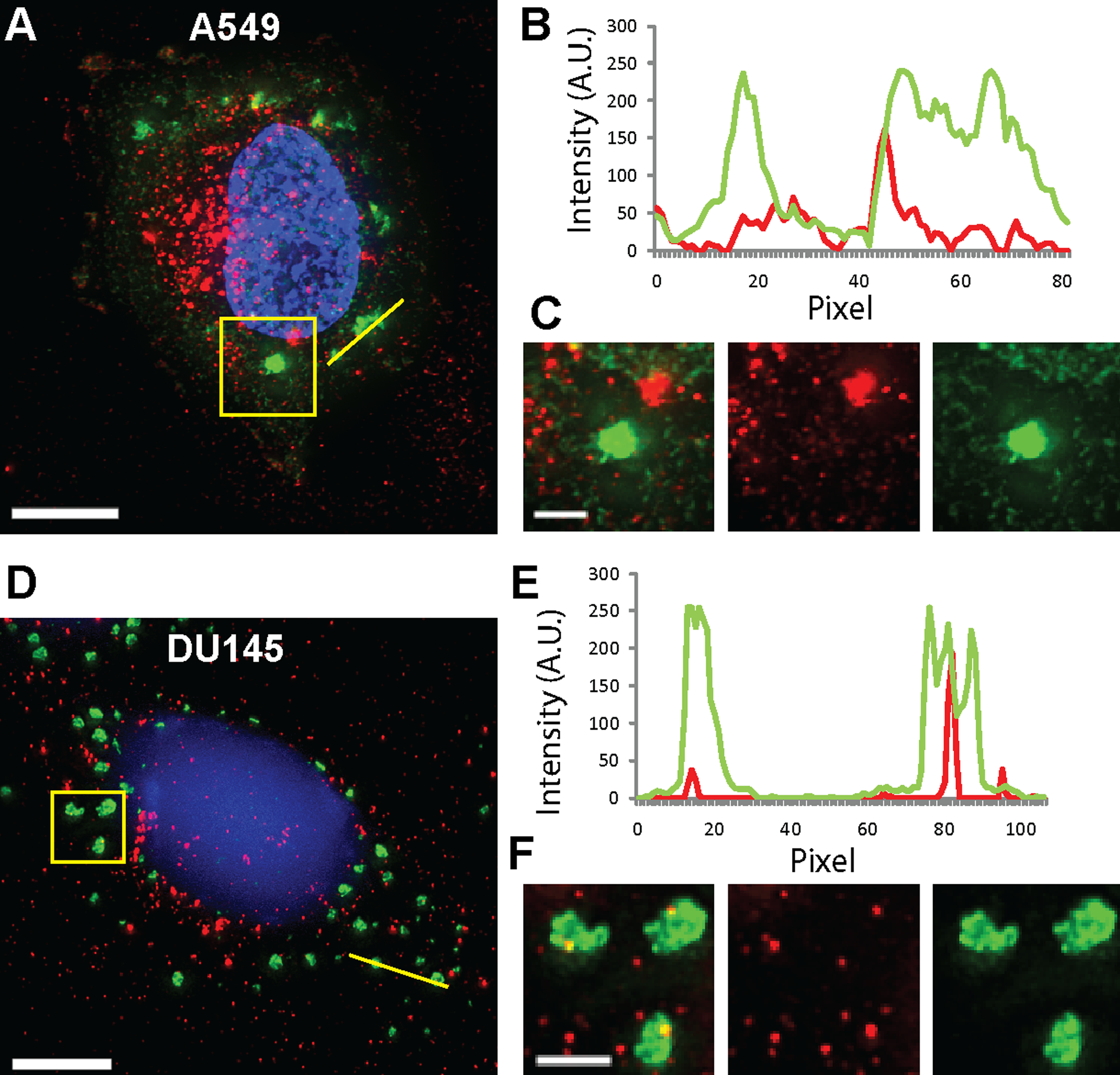

Supplement: Figure S4 — β-actin mRNAs interact with SGs during the stress response in A549 and DU145 cells. β-actin mRNA granules (red) interacted with G3BP or TIAR-stained SGs (green) in A549 (A) and DU154 cells (D) as demonstrated by intensity profiles along yellow lines (B and E) and insets displaying magnification of boxed areas (C and F). Nuclei were stained with DAPI. Scale bars, 10 µm and inset scale bars, 2.5 µm. (TIF) [file pone.0019727.s004.tif]

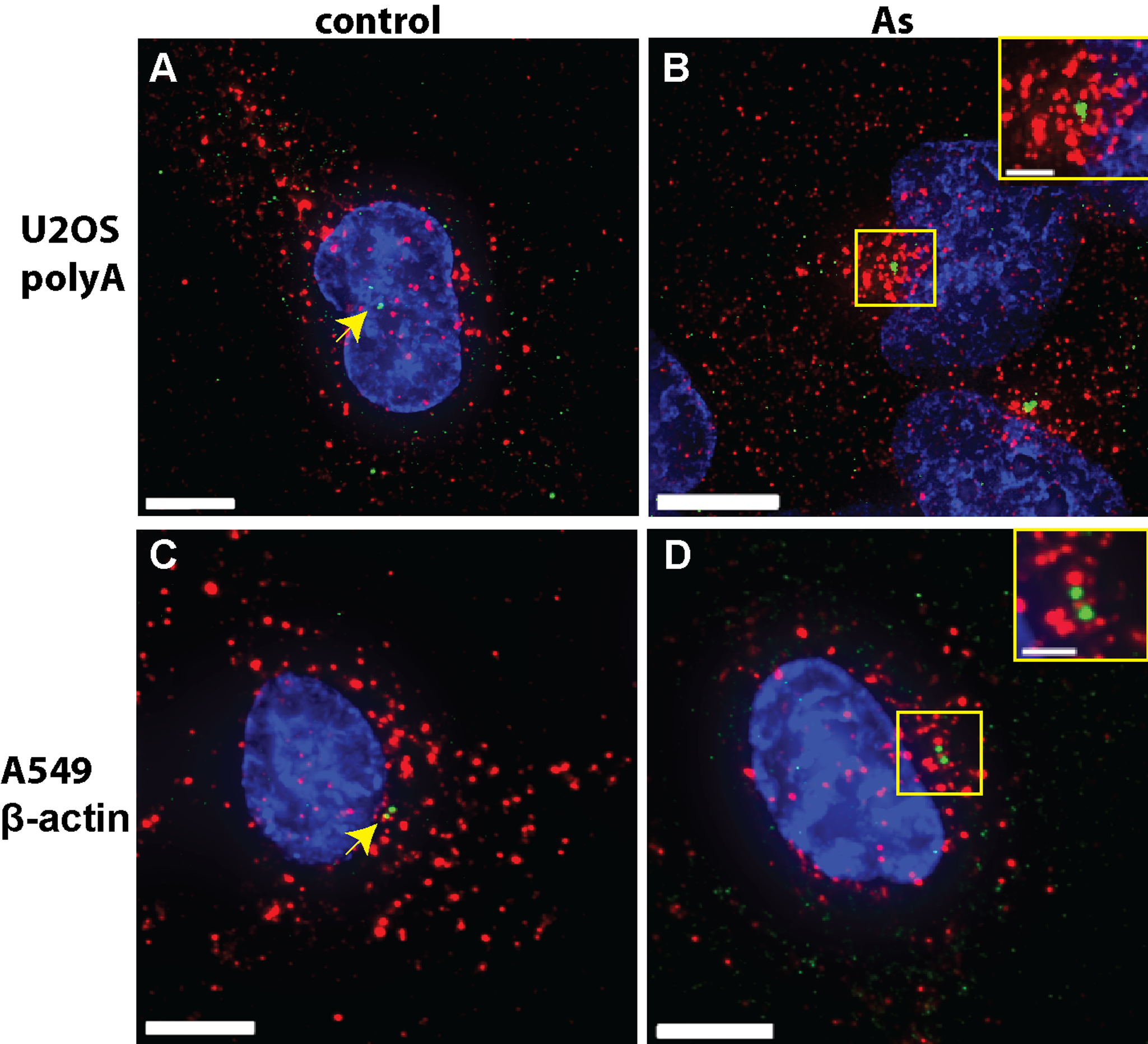

Supplement: Figure S5 — mRNAs localization near MTOC is a general mechanism during the stress response. Poly A+ (A) and β-actin (C) mRNA granules (red) are distributed in the cytoplasm in untreated U2OS cells and A549 cells, and localize near the MTOC (stained with a γ-tubulin antibody, green) after treatment with sodium arsenite (As) (B and D). Such mRNA localization was observed in 80% of U2OS and 66% of A549 respectively (data not shown). No colocalization between mRNAs and the MTOC is observed as indicated in the figures insets. In panels A and C the MTOC is indicated by the arrowhead. Nuclei were stained with DAPI. Scale bars, 10 µm and inset scale bars, 2.5 µm. (TIF) [file pone.0019727.s005.tif]

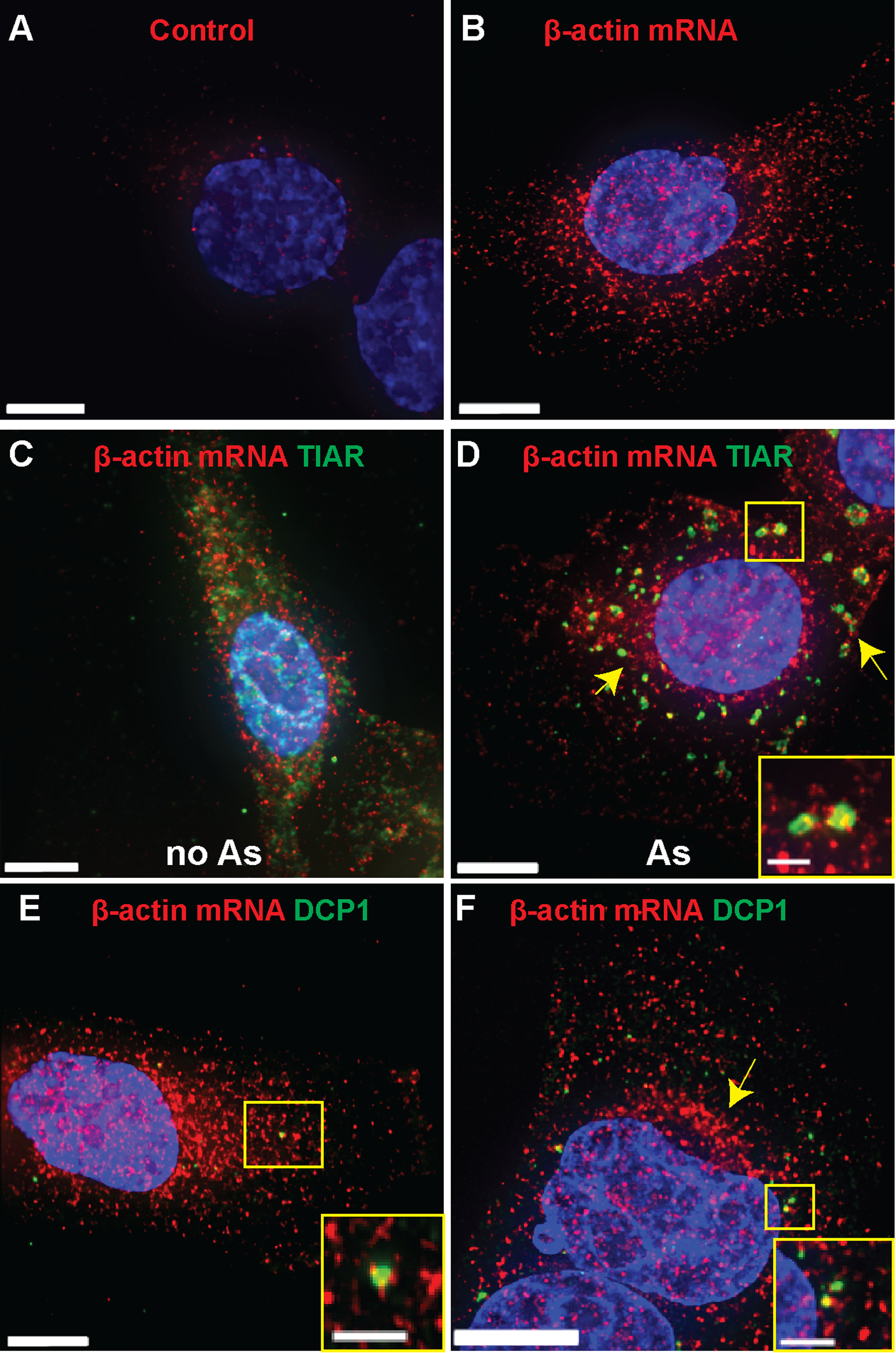

Supplement: Figure S6 — Specific detection of β-actin mRNA using FISH and interactions with SGs and PBs. Cells were hybridized with scrambled probes (A) or with linear probes targeting β-actin mRNA (B) as described in Material and Methods using similar exposure times (303 ms) and contrast enhancement. β-actin mRNA distribution in U2OS without (C and E) and with (D and F) sodium arsenite observed using FISH and immunofluorescence. Interactions with SGs and PBs are demonstrated by insets displaying magnification of boxed areas. β actin mRNAs along the cell edge and near the nucleus are indicated by the arrows. Scale bars, 10 µm and inset scale bars, 2.5 µm. Nuclei were stained with DAPI. (TIF) [file pone.0019727.s006.tif]

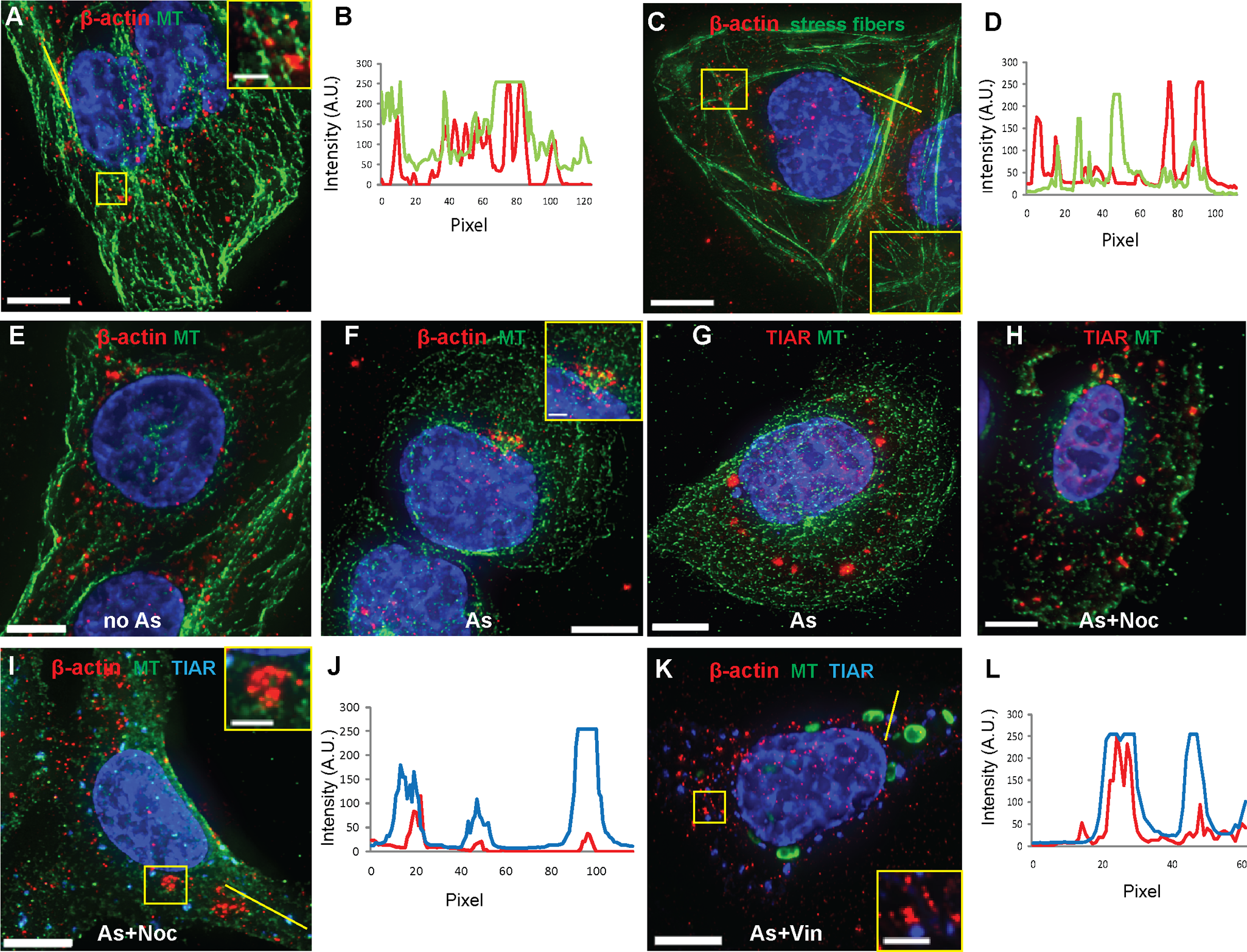

Supplement: Figure S7 — β-actin mRNA interaction with microtubules and stress fibers and effect of microtubule disruption. In unstressed U2OS cells, β-actin mRNA (ACTB) granules (red) colocalized with microtubules (MT) (A) and stress fibers (C) as shown by insets displaying magnification of boxed areas and profiles along yellow lines (B and D). (E and F) Upon sodium arsenite (As) treatment, β-actin mRNA granules colocalization with α-tubulin stained microtubules near the nucleus increases, as shown by inset displaying magnification of boxed area. (G and H) Treatment with nocodazole (Noc) disrupted microtubules (green) and impaired formation of arsenite mediated SG (red). Effect of nocodazole in A549 cells (I), or of vinblastin (Vin) in U2OS cells (K) in the presence of sodium arsenite. mRNAs (red) remained distributed in the cytoplasm, where they formed clusters (insets), and interacted with small SGs (blue), as seen by the profiles along yellow lines (J and L). Disruption of microtubules was demonstrated by staining for α-tubulin (green). Nuclei were stained with DAPI. Scale bars, 10 µm and inset scale bars, 2.5 µm. (TIF) [file pone.0019727.s007.tif]

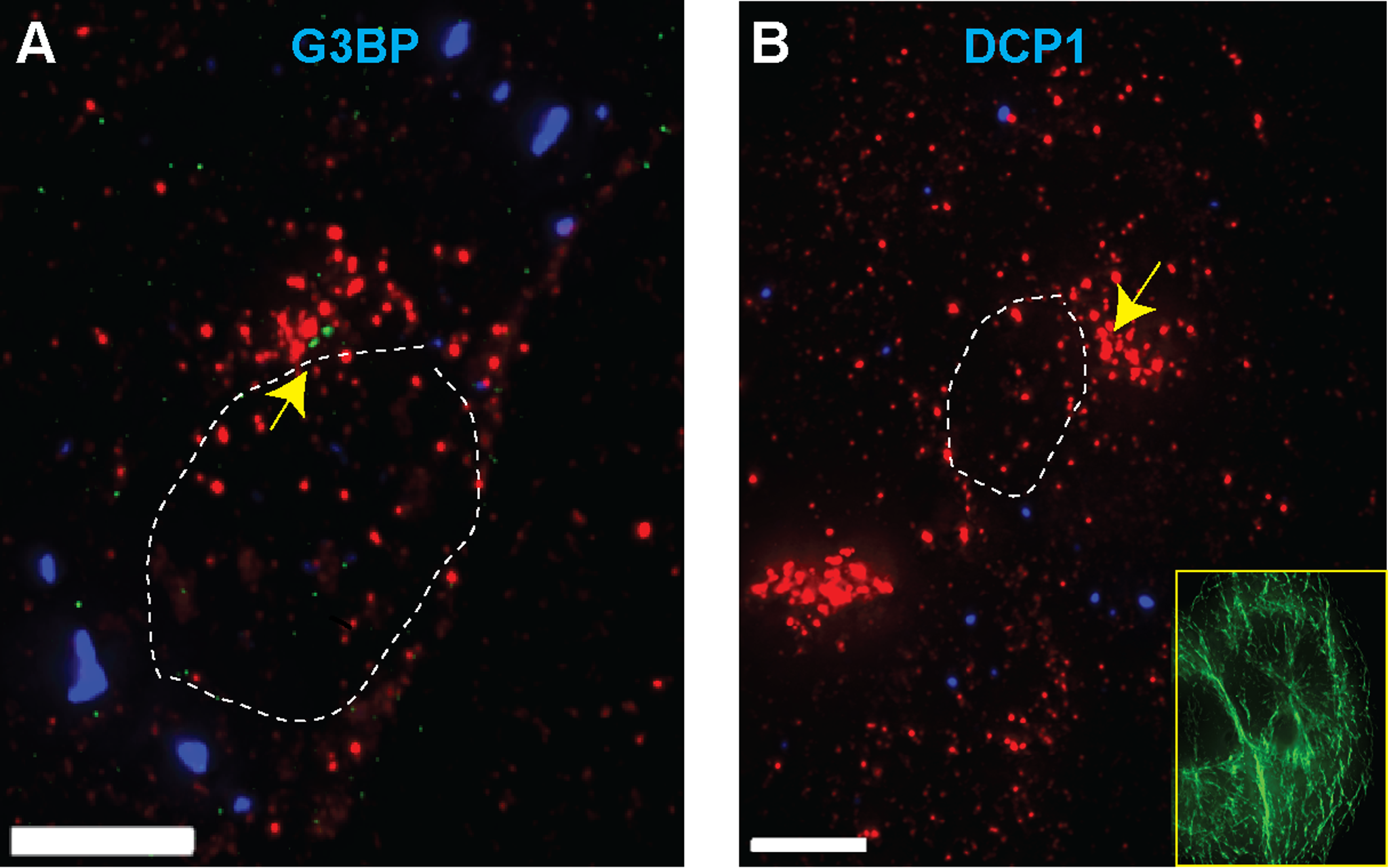

Supplement: Figure S8 — SGs and PBs do not localize near the MTOC during the stress response. G3BP-stained SGs (A) and DCP1-stained PBs (B) do not colocalize with the mRNAs near the MTOC. In (A) the MTOC was stained with a γ-tubulin antibody (green) and indicated by the arrow while in (B) the position of the MTOC, indicated by the arrow, was assessed by staining with an α-tubulin antibody (inset). Nuclei position is indicated by dotted line. Scale bars, 10 µm. (TIF) [file pone.0019727.s008.tif]

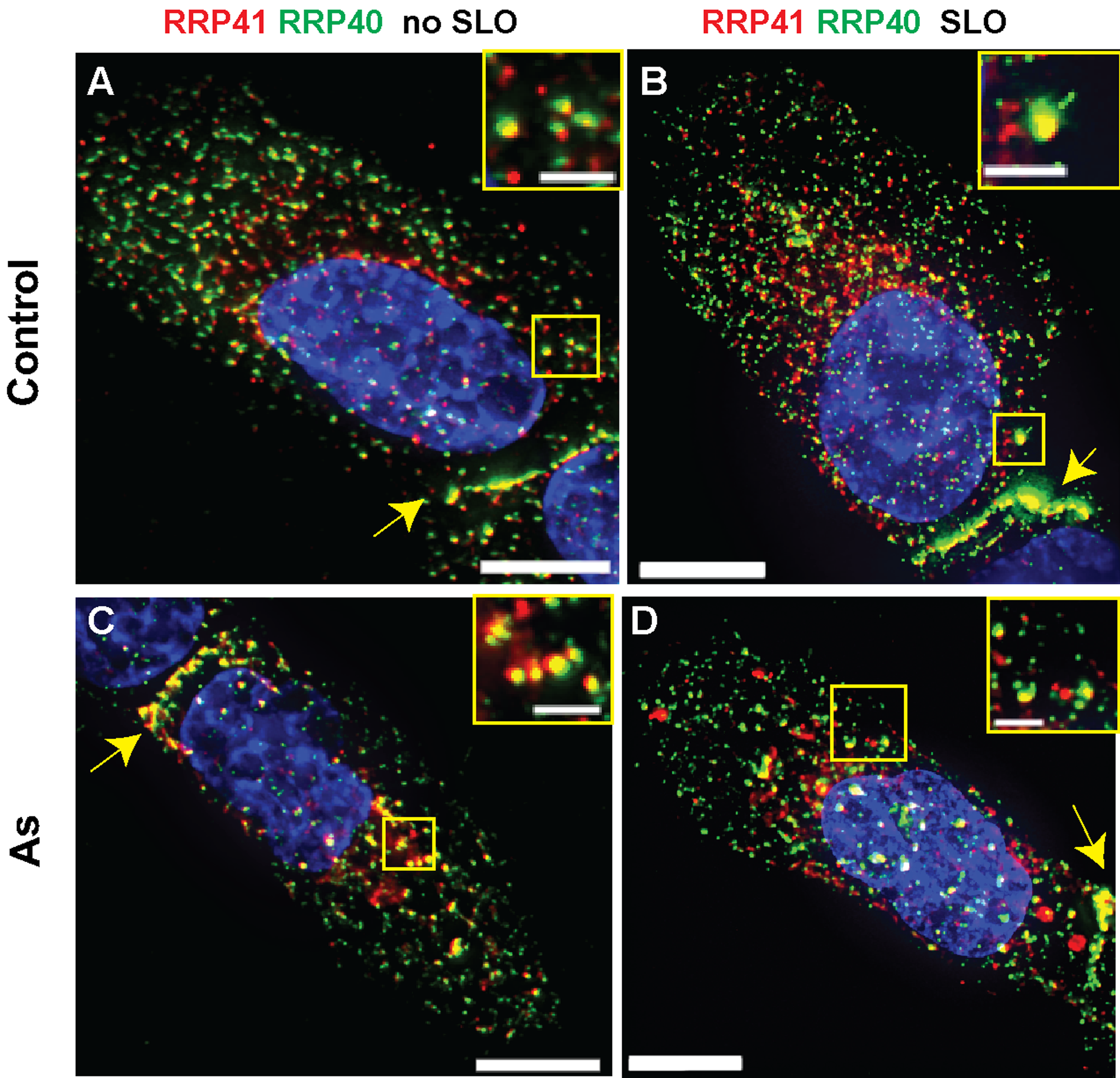

Supplement: Figure S9 — Distribution of exosome subunits-enriched granules in the cytoplasm of U2OS cells. Colocalization of RRP40 and RRP41 in untreated cells (A and B) and treated with sodium arsenite (C and D) along cells edges (arrows) and in cytoplasmic granules as demonstrated by insets displaying magnification of boxed areas. The overall distribution of the granules is not altered by SLO treatment. Nuclei were stained with DAPI. Scale bars, 10 µm and inset scale bars, 2.5 µm. (TIF) [file pone.0019727.s009.tif]
