# Supplementary material for: Characterizing mRNA Interactions with RNA Granules during Translation Initiation Inhibition
Source: PLoS One. 2011 May 5;6(5):e19727. doi: 10.1371/journal.pone.0019727 (PMC3088712; doi:10.1371/journal.pone.0019727)
Supplement: Table S1 — Sequences of MTRIPs targeting â-actin mRNA (ACTB) and the polyA tail with location within transcript. (DOC) [file pone.0019727.s010.doc]

**Table S1: Sequences of MTRIPs targeting β-actin mRNA (ACTB) and the polyA tail with location within transcript.**

| **Human β-actin** **mRNA target** | **Ligand** | **Location within transcript** |
| --- | --- | --- |
| PolyA+ Probe | 5*'*-biotin-TXTTTT**U**X**UUUUUU**X**UUUUUU**X**UU**-3’ |  |
| ACTB Probe 1 | 5*'*-biotin-TXTTTX**A**X**AGCACAGCC**X**GGA**X**A-**3*'* | 494-478 |
| ACTB Probe 2 | 5*'*-biotin- TTTTTTX**AUU**X**CCCGC**X**CGGCCG**X**G**-3*'* | 696-679 |
| ACTB Probe 3 | 5*'*-biotin-TXTTTTX**UCCUG**X**AACAA**X**GCAUC**XC-3*'* | 1479-1461 |
| ACTB Probe 4 | 5*'*-biotin-TXTTTTX**AAGCCC**X**GGC**X**GCC**X**CCA**-3*'* | 1748-1730 |
| ACTB Probe 5 | 5*'*-biotin-TTTTTX**GU**X**CAA**X**GGGG**X**ACUUCA**GGGX-3*'* | 290-269 |
| ACTB Probe 6 | 5*'*-biotin-TTTTTX**GCCAG**XGGX**ACGACCA**GA**GGCA**X-3*'* | 524-501 |
| hRSV Probe | 5*'*-biotin-**U**XTXTTX**AAAAA**X**GGGGCAAA**X**AA**-3*'* | 39-55;590-606;2323-2339 |
|  | Boldface: 2*'*-O-Methyl RNA; X: dT-C6-NH_2_; all others are DNA; underline: binding region |  |
