# Supplementary material for: Characterizing mRNA Interactions with RNA Granules during Translation Initiation Inhibition
Source: PLoS One. 2011 May 5;6(5):e19727. doi: 10.1371/journal.pone.0019727 (PMC3088712; doi:10.1371/journal.pone.0019727)
Supplement: Table S2 — SGs occupancy by â-actin mRNA in U2OS cells treated with increasing sodium arsenite concentrations. (DOC) [file pone.0019727.s011.doc]

**Table S2: SGs occupancy by β-actin** mRNA in U2OS cells treated with increasing sodium arsenite concentrations.

|  | ***0.5 mM As*** | ***1mM As*** | ***2 mM As*** |
| --- | --- | --- | --- |
| ***% SGs*** | **93** | **86** | **80** |
| ***% SG vol*** | **52±20** | **43±18** | **34±20** |

Percentage of SGs (% SGs) and percentage of SG volume (% SG vol) occupied by β-actin mRNA granules in the presence of sodium arsenite (As) 0.5, 1 and 2 mM. Standard deviations values are indicated.
